# Akirin Links Twist-Regulated Transcription with the Brahma Chromatin Remodeling Complex during Embryogenesis

**DOI:** 10.1371/journal.pgen.1002547

**Published:** 2012-03-01

**Authors:** Scott J. Nowak, Hitoshi Aihara, Katie Gonzalez, Yutaka Nibu, Mary K. Baylies

**Affiliations:** 1 Program in Developmental Biology, Sloan Kettering Institute, New York, New York, United States of America; 2 Cell and Developmental Biology, Weill Cornell Medical College, New York, New York, United States of America; 3 Weill Cornell Graduate School of Biomedical Sciences, Cornell University, New York, New York, United States of America; University of California San Francisco, United States of America

## Abstract

The activities of developmentally critical transcription factors are regulated via interactions with cofactors. Such interactions influence transcription factor activity either directly through protein–protein interactions or indirectly by altering the local chromatin environment. Using a yeast double-interaction screen, we identified a highly conserved nuclear protein, Akirin, as a novel cofactor of the key *Drosophila melanogaster* mesoderm and muscle transcription factor Twist. We find that Akirin interacts genetically and physically with Twist to facilitate expression of some, but not all, Twist-regulated genes during embryonic myogenesis. *akirin* mutant embryos have muscle defects consistent with altered regulation of a subset of Twist-regulated genes. To regulate transcription, Akirin colocalizes and genetically interacts with subunits of the Brahma SWI/SNF-class chromatin remodeling complex. Our results suggest that, mechanistically, Akirin mediates a novel connection between Twist and a chromatin remodeling complex to facilitate changes in the chromatin environment, leading to the optimal expression of some Twist-regulated genes during *Drosophila* myogenesis. We propose that this Akirin-mediated link between transcription factors and the Brahma complex represents a novel paradigm for providing tissue and target specificity for transcription factor interactions with the chromatin remodeling machinery.

## Author Summary

The proper development of the diverse array of cell types in an organism depends upon the induction and repression of specific genes at particular times and places. This gene regulation requires both the activity of tissue-specific transcriptional regulators and the modulation of the chromatin environment. To date, a complete picture of the interplay between these two processes remains unclear. To address this, we examined the activity of the evolutionarily conserved transcription factor Twist during embryogenesis of *Drosophila melanogaster*. While Twist has multiple activities and roles during development, a direct link between Twist and chromatin remodeling is unknown. We identified a highly conserved protein, Akirin, as a link between Twist and chromatin remodeling factors. Akirin is required for optimal expression of a Twist-dependent target during muscle development via interactions with the *Drosophila* SWI/SNF chromatin remodeling complex. Interestingly, Akirin is not required for activation of all Twist-dependent enhancers, suggesting that Akirin refines Twist activity outputs and that different Twist-dependent targets have different requirements for chromatin remodeling during development. Our data further suggests that Akirin similarly links the SWI/SNF chromatin remodeling complex with other transcription factors during development. This work has important ramifications for understanding both normal development and diseases such as cancer.

## Introduction

A fundamental question in embryonic development is how diverse cell lineages are specified, patterned and organized from a single common progenitor. These processes are governed by distinct gene expression programs administered by tightly regulated transcription factor activities during embryonic development. One mechanism whereby transcription factor activities are modulated is through direct interactions with cofactors. In addition, links between transcription factors and the general transcription machinery can be indirect, such as the actions of secondary factors that recruit chromatin remodeling complexes to modify the local chromatin environment and allow gene activation. The identification of these secondary effectors and their *in vivo* function is critical for understanding the regulation of transcription factor activity during embryonic development. Such interactions have significant ramifications for developmental disorders and diseases such as cancer.

The Twist transcription factor represents an ideal model for studying the regulation of transcription factor activity throughout development. Twist is a highly conserved transcription factor that is a key regulator of many developmental programs during embryogenesis as well as cancer metastasis [1]–[3]. In *Drosophila melanogaster*, Twist regulates multiple, discrete steps of mesoderm development, including gastrulation, segregation of populations of mesodermal cells, establishment and formation of the somatic musculature, and finally establishment of the adult musculature [4]–[7]. To achieve these diverse activities, Twist regulates a large number of target genes temporally within a discrete lineage resulting in a diverse array of outputs during mesodermal development [8]. A key question is how these diverse outputs of Twist activity during development are achieved.

The varied roles of Twist during different phases of embryonic development and the large number of Twist-regulated target genes suggest complex regulation of Twist activity [4], [8], [9]. Twist activity is often modulated by interactions with other transcription factors [10], [11]. Twist is a basic Helix-loop-Helix (bHLH) transcription factor that can homodimerize or heterodimerize, with distinct activities for each dimer pair. For example, Twist homodimers are responsible for activating target genes that direct cells to the somatic myogenic lineage [10]. By contrast, heterodimers between Twist and another bHLH protein, Daughterless, repress the somatic myogenic lineage [10], [11]. Twist activity is also regulated by interactions with other transcription regulators that bind closely linked DNA regulatory elements. One such interaction, with the NF-κB orthologue Dorsal, produces synergistic rather than cooperative activation of Dorsal targets during development [12]–[14]. Hence, Daughterless and Dorsal are examples of transcription regulators that physically interact with Twist to modify its output.

Other mechanisms that regulate Twist activity during development are less clear. One mechanism whereby transcriptional activity is indirectly regulated is through the action of chromatin modifying factors that generate a local chromatin environment that allows and/or favors the expression of a specific target gene. The structure of chromatin is modified by two predominant mechanisms: the marking of nucleosome tails with post-translational modifications such as acetylation, phosphorylation and methylation, and the remodeling of the local chromatin environment via factors that reposition nucleosomes in a local gene environment [15]. Nucleosome repositioning occurs via the activity of ATP-dependent chromatin remodeling complexes such as the Brahma-containing (BRM) complex, the *Drosophila* orthologue of the yeast SWI/SNF chromatin remodeling complex [15], [16]. Mutations in BRM complex subunits have revealed essential roles for BRM in such processes as homeotic gene expression, oogenesis, and cell cycle control [17]–[22]. Loss-of-function studies have determined that BRM-regulated chromatin remodeling activity is required for most RNA Polymerase II-regulated transcription [23]. Most eukaryotic organisms have two different compositions of the SWI/SNF complex; in *Drosophila*, these complexes are designated the BAP (Osa-containing) and PBAP (polybromo/Bap180, Bap170 and SAYP-containing) complexes [15], [16], [24]. Both BAP and PBAP are linked to gene activation and repression, are present in the same cells, and perform unique yet cooperative functions during development [19], [24]–[28]. Both the association of BRM complexes with transcription factors and BRM complex targeting and regulation during embryogenesis remain an area of considerable interest. Moreover, links between BRM complexes, chromatin remodeling and Twist have not yet been elucidated.

The nuclear protein Akirin was shown to regulate gene expression in several different transcription pathways, yet its mechanism of action remained unclear [29], [30]. Here we identify Akirin as a factor that facilitates an interaction between Twist and the BRM chromatin remodeling complex to promote gene expression. We find that Akirin interacts both physically and genetically with Twist at Twist-dependent enhancer regions and positively regulates expression of *Dmef2*, a Twist-regulated gene that is critical for somatic myogenesis during development. As would be predicted by this interaction, *akirin* mutant embryos show a range of somatic muscle phenotypes. Akirin is widely associated with regions of active transcription. We find that Akirin colocalizes with subunits of the BRM chromatin remodeling complex on polytene chromosomes and interacts genetically with core subunits of the BRM complex during myogenesis. Finally, we verify with chromatin immunoprecipitation that Twist, Akirin, and a core subunit of the BRM complex all occupy the Twist-dependent *Dmef2* enhancer. Curiously, Akirin/Twist interactions are not required at all Twist-dependent enhancers, as evidenced by our chromatin immunoprecipitation experiments at the *eve* MHE. These results suggest that Akirin functions as a BRM accessory protein that links the BRM chromatin remodeling machinery to Twist transcription factor activity at a subset of Twist-regulated enhancers. These results provide a common mechanism by which Akirin links chromatin remodeling factors to spatiotemporal-specific gene activation.

## Results

### Akirin is a conserved Twist-interacting nuclear protein

To identify proteins that interact with Twist, we performed a yeast double interaction screen [31], [32]. Two Twist-regulated enhancers from the *Dmef2* [33] and *tinman* [34] genes were cloned upstream of a *HIS3* reporter. Yeast strains containing these enhancer/reporter constructs were first transformed with a Twist expression vector and then with a 0–6 hr embryonic cDNA library fused to a Gal4 activation domain. Transformants were positively scored for cDNAs that activated *HIS3* expression at *Dmef2*, *tinman*, or both enhancers in a Twist-dependent manner. We identified 28 cDNAs that activated expression from both enhancers. From this group, we selected the then-unknown gene *CG8580* for further study. *CG8580* was found to require the presence of Twist for activation of the *HIS3* reporter [32].

We initially named *CG8580* as *bhringi* [32], but recent groups have re-designated this gene and its orthologues as *akirin* [29], [35]. For clarity, we have adopted this nomenclature. The *Drosophila akirin* gene encodes a highly conserved 201-residue protein with orthologues present in over 24 different metazoan genomes [35]. Aside from a predicted nuclear localization sequence, *Drosophila* Akirin has no known protein motifs [35]. We raised antibodies against a peptide sequence found in the N-terminal region of *Drosophila* Akirin (Figure S1). Immunofluorescence staining of embryos at various stages of development showed that Akirin is ubiquitously expressed and found in the nuclei of all cells of the developing embryo (Figure S2).

### *akirin* mutants display a range of muscle phenotypes

**Figure 1:**
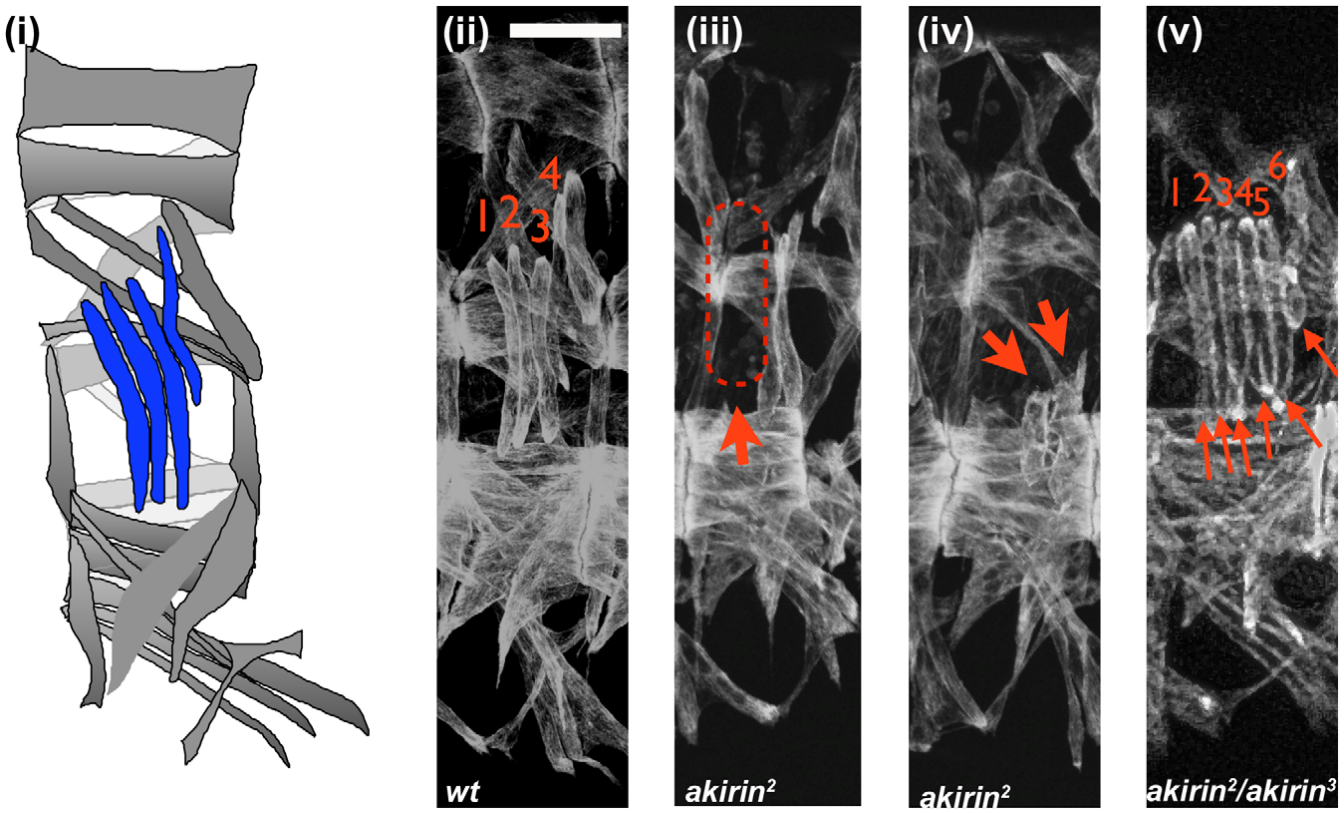
Akirin mutants display a range of muscle phenotypes. Lateral views of stage 16 wild-type (ii, *wt*) and *akirin* mutant (iii, iv, v) embryos stained with anti-tropomyosin antibodies demonstrate the types of muscle phenotypes observed. All allelic combinations are listed as maternal/paternal contribution. In all figures, anterior is to left and dorsal is up, scale bar = 25 µm. Lateral transverse (LT) muscles are used to illustrate the following predominant muscle defects observed in *akirin* mutants (red arrows): (iii) missing muscles, (iv) improperly attached muscles, and (v) duplicated muscles. Schematic of wild-type muscle pattern is shown in (i). Penetrance of each defect is given in Table 1.

The somatic muscles of *Drosophila* embryos are arranged in a stereotypic, repeated pattern of 30 muscles within each abdominal hemisegment (Figure 1) [36]. Because Akirin interacted with Twist, a transcription factor that is essential to the proper patterning of the somatic musculature, we examined the somatic musculature of *akirin* mutant embryos, using publicly available and newly generated *akirin* alleles (See Materials and Methods and Figure S3). *akirin* mutant embryos exhibit a range of somatic body wall muscle phenotypes. Three classes of defects were observed: missing muscles, attachment defects, and duplicated muscles (Figure 1). While these defects were seen easily in the 4 lateral transverse (LT) muscles, the same classes of defects were detected in several other muscles, particularly the DT1, DO3, DO4 and DA3 muscles. Misattached, missing or duplicated muscles were found in stage 16 (15 h after egg laying (AEL)) *akirin^2^* homozygous mutant embryos (46.5%, n = 114 embryos), and embryos from crosses of *akirin^2^* females with *akirin^3^* males (67%, n = 52 embryos), or *akirin^3^* females with *akirin^5^* (44.7%, n = 38 embryos) males (Table 1). The low penetrance of muscle phenotypes seen in *akirin* mutants is likely due to the high degree of maternal loading of *akirin* RNA (Figure S3). We attempted to generate *akirin* maternal/zygotic mutants by making germline clones using *akirin^3^* mutants [37]. However, no *akirin^MZ^* mutant embryos were recovered, suggesting that Akirin is important for oocyte formation (data not shown). Therefore, homozygous viable *akirin^2^* females likely produce embryos that are more highly sensitized to low levels of *akirin* mRNA in the oocyte. Importantly, the missing muscle phenotype is not due to a failure to properly specify muscle founder cells, as immunohistochemistry of founder cell identity proteins, including Slouch, Even-skipped and Krüppel, did not show a decrease in the numbers of founder cells in *akirin* mutants (Figure S4). Also, in *akirin* mutant embryos there were no obvious signs of muscle degeneration or late differentiation defects [38] (data not shown). Epidermal development was normal, and the cuticle developed normally (data not shown).

**Table 1:** Penetrance of muscle phenotypes in *akirin* mutant embryos.

Genotype	Embryos examined	Penetrance^a^	Missing muscles^b^	Attachment defects^b^	Duplicated muscles^b^
*akirin^2^*	114	46.5%	67.9%	83.0%	18.9%
*akirin^2^/akirin^3^*	52	67.0%	71.4%	68.5%	25.7%
*akirin^3^/akirin^5^*	38	44.7%	35.0%	35.0%	6.0%
*wild-type*	85	2.3%	1.1%	1.1%	0.0%

a Penetrance indicates percentage of mutant embryos that have a defect in n≥2 hemisegments.

b Prevalence of missing, attachment, and duplication defects are given as percentage of observed defect in mutant embryos.

### Akirin interacts both genetically and physically with Twist

**Figure 2:**
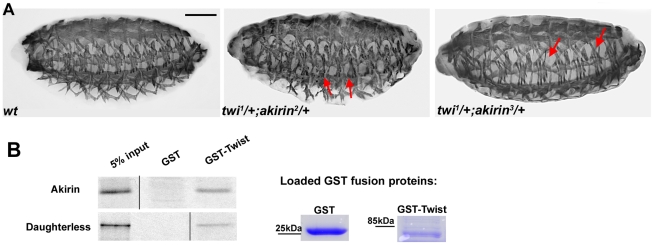
Akirin interacts both genetically and physically with Twist. (A) Lateral views of stage 16 wild-type (*wt*) and embryos heterozygous for both *twist* and *akirin* (*twi^1^/+; akirin^2^/+* and *twi^1^/+; akirin^3^/+*) stained with tropomyosin antibodies to visualize body wall muscles. Double heterozygous embryos have defects in muscle patterning (indicated by red arrows), suggesting a genetic interaction between *akirin* and *twist*. Scale bar = 50 µm. (B) GST pull-down experiments indicate that GST-Twist interacts physically with *in vitro* expressed Akirin protein. GST-Twist pull-down of *in vitro* translated Daughterless provided as positive control for pull-down. Loading of GST-fusion proteins, as verified by Coomassie staining is provided. Note that (B) is a composite figure; lanes were omitted from the same gel for sake of clarity and direct comparison.

The muscle phenotypes in *akirin* mutant embryos were reminiscent of those observed in embryos in which *twist* expression is modified by RNAi in the during muscle fiber formation [11]. To determine if *akirin* interacts genetically *in vivo* with *twist*, we examined the patterning of the somatic body wall musculature in embryos that are heterozygous for both *twist* and *akirin*. While *twi^1^*/+ embryos [39], *akirin^2^/+* and *akirin^3^/+* embryos have a wild-type somatic muscle pattern (data not shown), stage 16 embryos that are heterozygous for both *twist* and *akirin^2^* and *akirin^3^* (*twi^1^/+; akirin^2^/+*, and *twi^1^/+; akirin^3^/+*) show a general disruption of the muscle pattern, with missing and misattached muscles (*twi^I1^/+; akirin^2^/+* : 21.6% of 60 embryos examined and *twi^1^/+; akirin^3^/+*: 18.5% of 105 embryos examined, Figure 2A).

Additionally, *in vitro*-translated Akirin was successfully pulled down using a GST-Twist fusion protein indicating a physical interaction between Akirin and Twist (Figure 2B). Taken together, these data confirmed our initial results of the yeast double interaction screen and demonstrated both a physical and functional interaction between Akirin and Twist.

### Dmef2 enhancer activity is reduced in *akirin* mutant embryos

**Figure 3:**
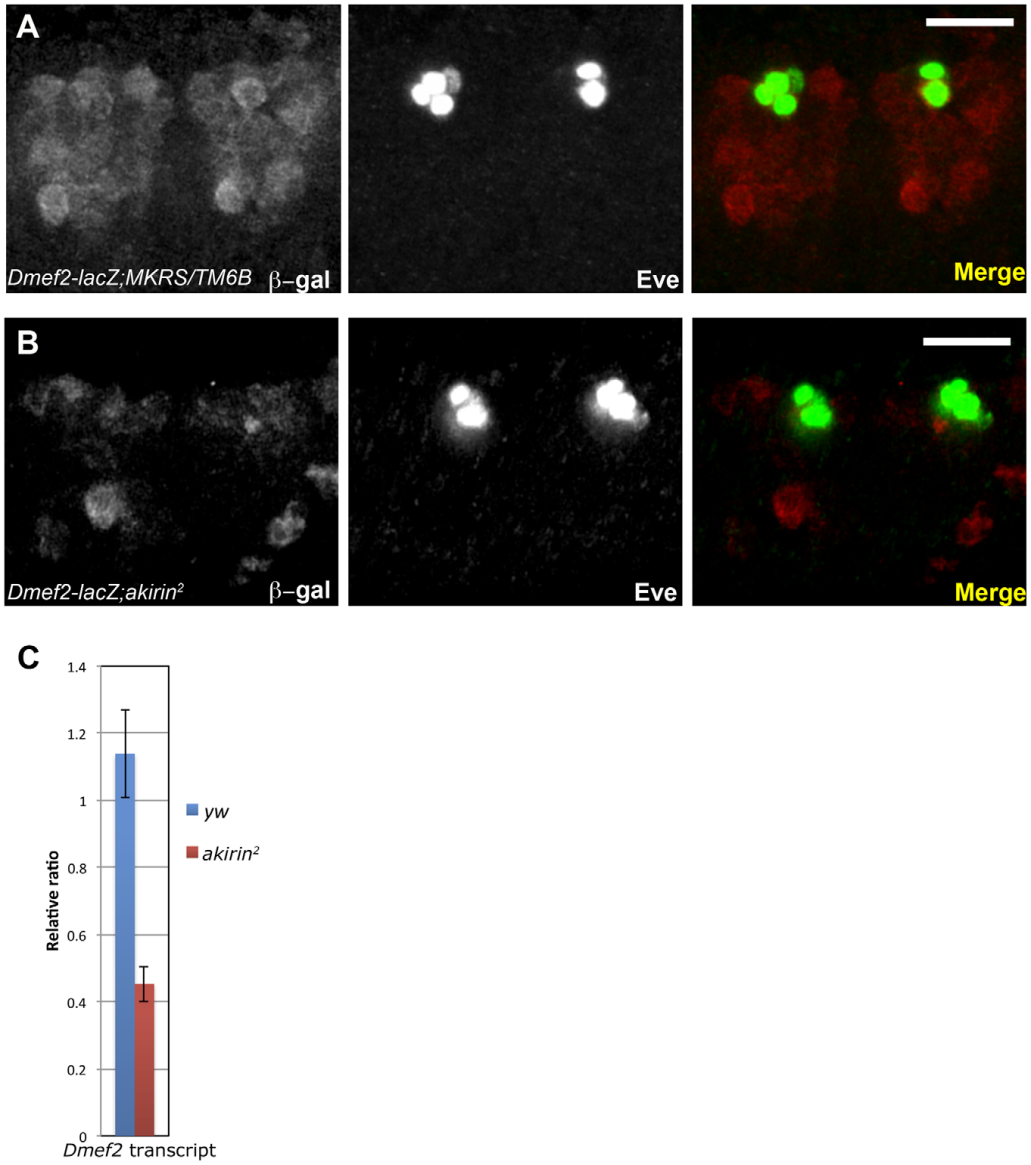
*Dmef2* enhancer activity is reduced in *akirin* mutants. Confocal micrographs of two hemisegments of transgenic late stage 11 wild-type (A) and *akirin^2^* homozygous embryos (B) containing the *Dmef2-lacZ* reporter construct [33] showing immunoreactivity to β-galactosidase (red) and Even-skipped (Eve, green). *Dmef2-lacZ* reporter expression was reduced in *akirin^2^* mutants compared to wild-type embryos. Images of wild-type and *akirin^2^* embryos were acquired with identical exposure settings (see Materials and Methods). (C) RT-PCR of *Dmef2* mRNA isolated from 4–6 hour-old wild-type and *akirin^2^* mutant embryos demonstrates that *Dmef2* expression is reduced in *akirin^2^* mutants. Bar in (A) and (B) = 20 µm.

Akirin was initially identified in our screen as a Twist interacting protein in the context of the *Dmef2* enhancer. In addition, the muscle defects in *akirin* mutants are similar to the phenotypes in embryos with modified *Dmef2* or *twist* expression [7], [11], [40]–[42]. These data suggested that Akirin and Twist interact to positively regulate transcription from *Dmef2*. To test this *in vivo*, we analyzed *akirin* mutant embryos that carry a *lacZ* transgene regulated by the same *Dmef2* muscle enhancer [33] used in our initial double interaction screen. This particular enhancer requires Twist activity for early *Dmef2* expression during somatic myogenesis. Regulation of *Dmef2* by Twist is critical for the subsequent establishment of the somatic musculature [8], [33], [40], [41], [43]–[48]. Whole-mount antibody staining for β-galactosidase (β-gal) indicated that *akirin* mutants have reduced expression of β-gal compared to wild-type embryos with the same transgene at the same developmental stage (Figure 3A, 3B). Densitometric analysis of Western blotting supported the observation that total β-gal levels were changed in *akirin* mutant extracts relative to wild-type embryo extracts (Figure S5). To confirm that endogenous *Dmef2* expression levels were affected, we performed RT-qPCR analysis on total mRNA prepared from wild-type and *akirin^2^* mutant embryos at the same developmental age (4–6 h AEL). Accordingly, we observed a 2.75-fold reduction in *Dmef2* transcripts in *akirin^2^* mutant embryos compared to wild-type embryos at this time (Figure 3C).

For comparison, we examined a second Twist target gene, *even-skipped*. No reduction in protein levels of Even-skipped (Eve) or levels of expression from *eveMHE-lacZ* reporter constructs were observed in *akirin* mutant embryos at the same stage (Figure 3B and Figure S5). These data indicated that Twist and Akirin interact to positively regulate expression of the *Dmef2* reporter. These data also suggested that not all Twist target genes and/or their associated enhancer elements require Akirin activity for their proper expression.

### Akirin protein is detected at actively transcribed gene loci

**Figure 4:**
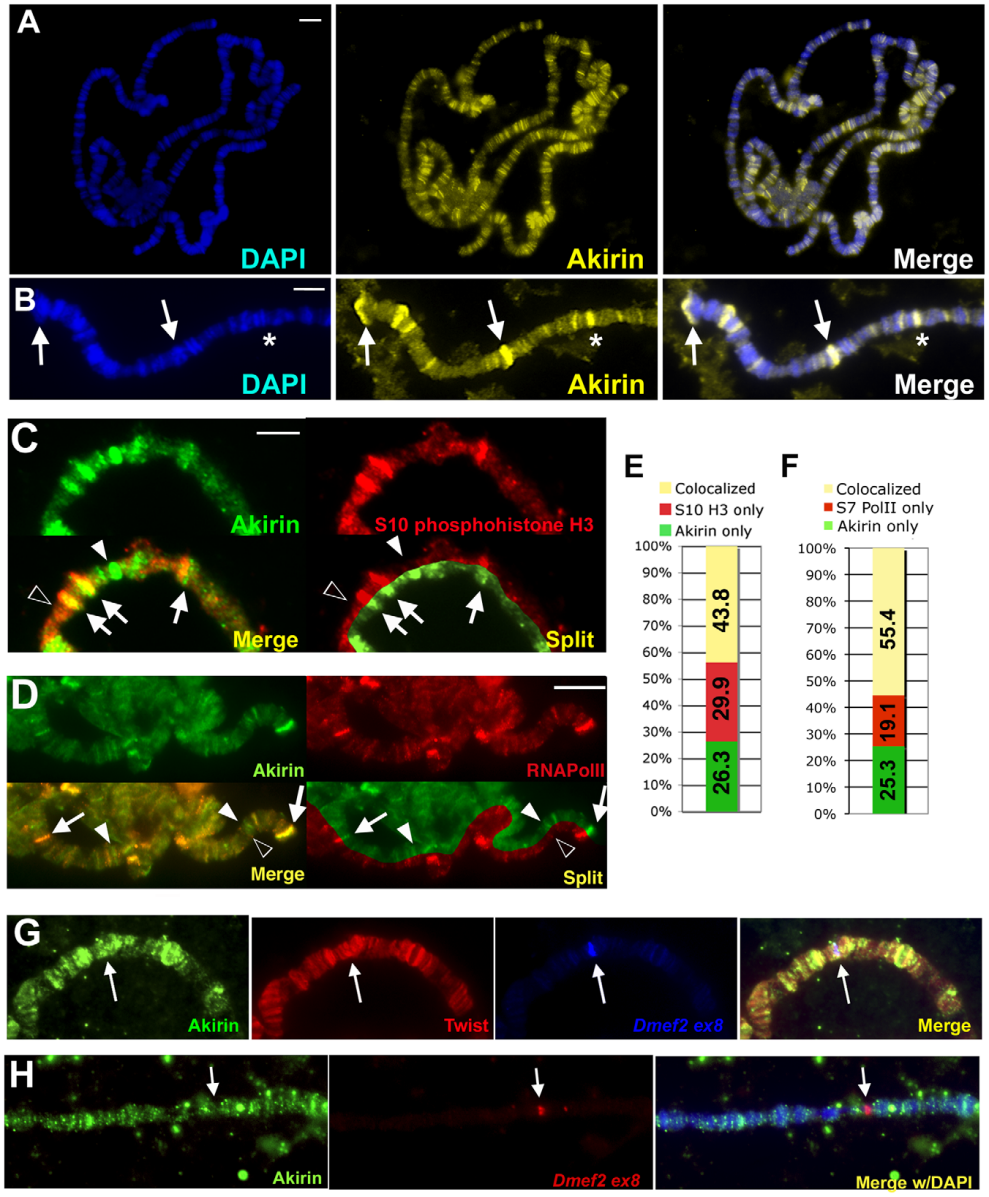
Akirin protein is broadly distributed throughout the genome. (A,B) Polytene chromosomes were prepared from wild-type larvae, immunostained with antibody against Akirin and counterstained with DAPI to visualize DNA. Akirin is distributed at many sites throughout the genome and found at both puffed (arrows in (B)) and non-puffed (* in (B)) regions. (C,D) Detail of wild-type polytene chromosomes immunostained with antibodies against Akirin (green) and (C) Ser10-phosphorylated histone H3 (red) or (D) Ser7-phosphorylated RNA Polymerase II (red) indicate that Akirin is found at some but not all regions of active transcription in polytene chromosomes. See Figure S6 for whole-genome squashes. Split presentation provided to compare banding pattern. White arrows indicate colocalization, white arrowheads indicated Akirin-only bands, black arrowheads indicate Akirin-negative bands. (E,F) Quantification of colocalization between Akirin and (C) Ser10-phosphorylated histone H3 and (D) Ser7-phosphorylated RNA Polymerase II. (G) Twist and Akirin colocalize at Twist targets in polytene chromosomes. Chromosome squashes were prepared from larvae expressing Twist under the control of the *Sgs3-GAL4* driver (*Sgs3-GAL4>UAS-twist*). Polytenes were hybridized with DNA probes against exon 8 of *Dmef2*, and further immunostained with antibodies against Akirin (green) and Twist (red). Both Twist and Akirin localize to the *Dmef2* locus (arrow) when Twist is expressed in salivary glands. (H) Akirin does not localize to the *Dmef2* locus in salivary glands that do not express Twist. Scale bar in all images = 5 µm.

In addition to our observed relationship with Twist, Akirin has been linked to other transcriptional regulators in various insect and mouse contexts [29], [30], [49]. However, in each of these contexts, the mechanism by which Akirin promotes gene expression remained unclear. Because Akirin does not have a consensus DNA-binding domain or predicted catalytic activity, we hypothesized that Akirin regulates gene expression through its interactions with transcriptional regulators and would therefore localize to regions of active transcription. We analyzed the distribution of Akirin on polytene chromosomes and found Akirin localization throughout the genome including puffed regions of polytene chromosomes (Figure 4A, 4B), which indicate active transcription [50]. For further confirmation, co-immunostaining for Akirin and Serine 10-phosophorylated histone H3, a histone modification that serves as a marker of actively transcribing loci in polytene chromosomes, was performed [51]–[53]. Akirin partially colocalized with Ser10-phosphohistone H3 staining (43.8% colocalization, see Figure 4C, 4E and Figure S6A). To strengthen these results, we performed additional co-immunostaining for Akirin and Ser7-phosophorylated RNA polymerase II [54], which also showed partial colocalization between these two stains (55.4%, see Figure 4D, 4F and Figure S6B). These data confirmed that Akirin is associated with some actively transcribing loci throughout the genome. In addition, because Twist is not expressed in wandering third instar salivary glands ([55] and data not shown), these data suggested that Akirin interacts with other transcriptional regulators for its function in this tissue. However, when we ectopically expressed Twist in the salivary glands using *Sgs3*-GAL4 [56], we found that Twist and Akirin partially colocalize on polytene chromosomes (54% colocalization, Figure 4G). Furthermore, under these conditions both Twist and Akirin colocalized at the *Dmef2* locus (Figure 4G), and Dmef2, which is not normally expressed in salivary glands, is now expressed (Figure S7). We note that Akirin is not localized to the *Dmef2* locus when Twist is not present (Figure 4H). Together, these data supported our earlier results indicating that Akirin acts with Twist to promote the expression of a Twist-regulated target. Finally, the colocalization of Akirin with regions of active gene expression in a tissue that does not normally contain Twist confirmed that Akirin functions as a general regulator of gene expression.

### Akirin colocalizes with components of the Brahma chromatin remodeling complex

**Figure 5:**
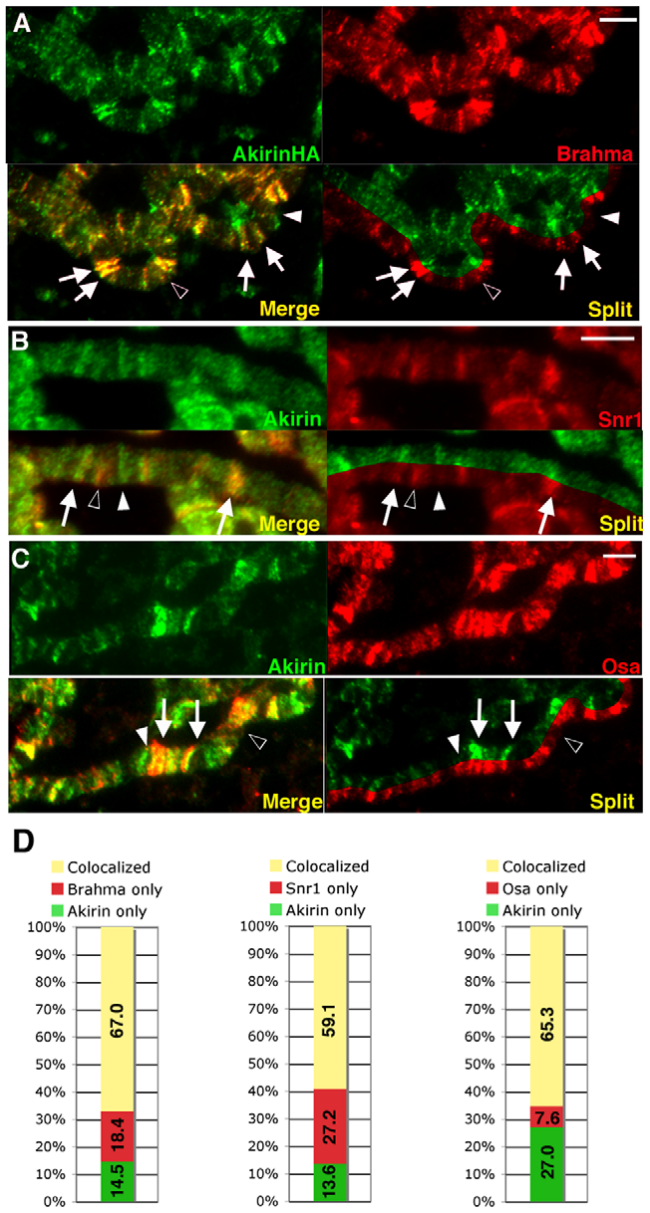
Akirin colocalizes with subunits of the SWI/SNF-class Brahma chromatin remodeling complex. (A–C) Detail of polytene chromosome squashes demonstrating immunocolocalization of Akirin with Brm complex subunits. See Figure S9 for whole genome squash images. (A) To avoid cross-reactivity between Akirin and Brahma primary antibodies, polytene chromosomes were prepared from larvae expressing HA-tagged Akirin (*UAS*-Akirin-HA) under control of the *Sgs3-GAL4* driver, and immunostained with antibodies against HA (green) and Brahma (red). Extensive colocalization was observed between Akirin-HA and Brahma (white arrows). Examples of Akirin-HA immunostain without Brahma colocalization (white arrowhead), and Brahma signal without Akirin colocalization (black arrowhead) are indicated. See Figure S8 for comparison of Akirin-HA and endogenous Akirin immunolocalization. (B,C) wild-type polytene chromosomes were immunostained with antibodies against Akirin (green) and Snr1 (B) or Osa (C). Examples of Akirin signal without corresponding Snr or Osa colocalization are indicated (white arrowhead), and Snr or Osa signal without corresponding Akirin immunostain (black arrowhead) are indicated. (D) Quantification of colocalization/non-overlap between Akirin and Brm complex subunits -positive polytene bands in (A–C). Scale bar in all images = 5 µm.

One mechanism whereby Akirin might function as a general cofactor for gene expression would be through interactions with chromatin remodeling complexes. A *Drosophila* whole-genome yeast 2-hybrid experiment [57] suggested that Akirin interacts with BAP60, a core subunit of the *Drosophila* SWI/SNF class Brahma (BRM) chromatin remodeling complex [58]. Immunostaining of polytene chromosomes with antibodies against the Brahma (Figure 5A) and Snr1 (Figure 5B) core subunits revealed that Akirin colocalized with BRM (67% and 59.1% colocalization respectively, Figure 5D and Figures S8, S9). Akirin also colocalized with Osa (65.3% colocalization), a subunit exclusive to the BAP complex (Figure 5C, 5D and Figure S9). These data therefore suggested that Akirin associates with BRM complex components but is not a core BRM complex subunit.

### Akirin interacts with BRM complex subunits during embryonic myogenesis

**Figure 6:**
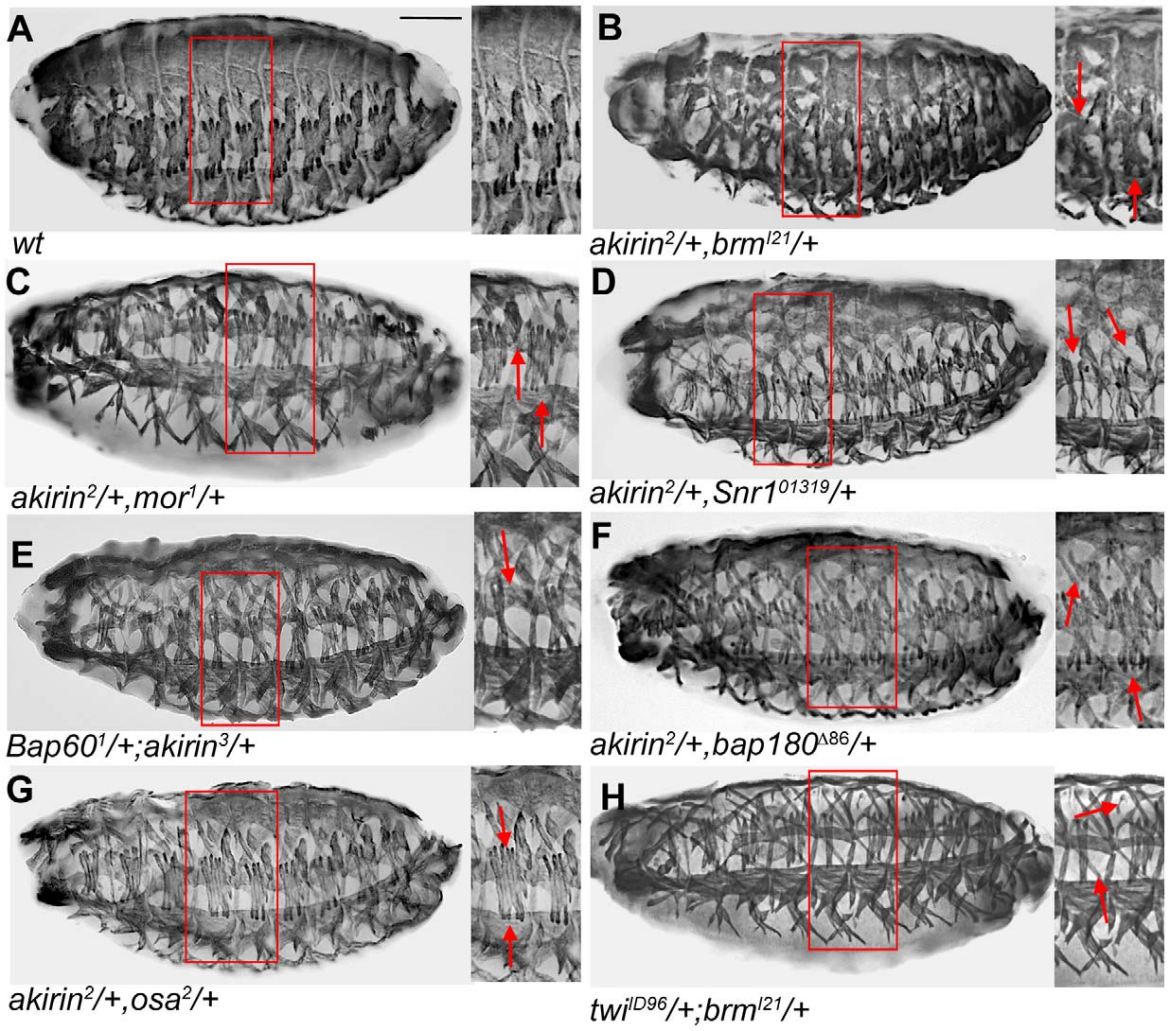
Akirin interacts genetically and physically with BAP and PBAP subunits during embryonic myogenesis. (A–G) Lateral views of stage 16 Wild-type (*wt*) and embryos heterozygous for both *akirin* and indicated subunits of BAP and PBAP complexes. All embryos stained to reveal somatic musculature with tropomyosin antibodies. Thin red boxes in whole embryo photos indicate region shown in higher magnification for each embryo. Double heterozygous embryos have defects in general muscle pattering, suggesting a genetic interaction between *akirin* and components of the BRM chromatin remodeling complex (See Table 2 for quantification of defects). (H) Lateral view of stage 16 embryo heterozygous for both *twist* and *brahma*. Muscle patterning defects observed in these embryos suggest a genetic interaction between *twist* and *brahma* (A,C). Scale bar in panels A–H = 50 µm.

Given the observed colocalization between Akirin and the Brahma, Snr1 and Osa BAP subunits on polytene chromosomes, we hypothesized that there is a functional association of these proteins during embryonic development. To test this, the patterning of the somatic muscles in stage 16 embryos that are double heterozygotes for both *akirin* and *brahma* was examined (Figure 6B). Both *brm^I21^/*+ and *akirin^2^/+* (single heterozygous) embryos have a normal somatic muscle pattern (Figure S10), but the *brm^I21^/*+,*akirin^2^/*+ double heterozygous embryos (35%, n = 55) displayed disruptions in the somatic muscles, with both missing and improperly attached muscles (Figure 6B, Table 2). We further analyzed embryos that are double heterozygotes of both *akirin* and other BRM complex core subunits (Figure 6, Table 2). Although heterozygous embryos for other BRM complex subunits do not show a somatic muscle phenotype (Figure S10), muscle phenotypes were observed in *akirin^2^/*+,*moira^1^/*+ (40%, n = 48, Figure 6C), *akirin^2^/*+,*Snr1^01319^/*+ (45%, n = 52, Figure 6D), and *Bap60^1^/*+;*akirin^3^/*+ embryos (40%, n = 55, Figure 6E). Additionally, *akirin^2^/+,bap180^Δ86^/+* (38%, n = 45, Figure 6F) and *akirin^2^/+,osa^2^/+* (42%, n = 52, Figure 6G) embryos also had disrupted muscle patterning. To ensure that these defects were specific for subunits of the BRM complex and not due to a general interaction between *akirin* and other chromatin factors, double heterozygous combinations of *akirin* with alleles of *Polycomb*, *Nurf-38*, *Iswi*, and *Su(var)3-9* were tested. Each of these allelic combinations showed muscle patterning defects in fewer than 5% of embryos. These results confirmed a functional interaction during muscle development between Akirin and core BRM subunits, as well as PBAP and BAP-specific subunits. Based on our data thus far, we hypothesized that Akirin mediates Twist-BRM interactions on a subset of Twist target genes. To begin to test this, we examined whether whether *twi* and *brm* genetically interact. We find that the somatic muscle pattern is disrupted in *twi^I1^/+; brm^I21^/+* double heterozygous embryos (23%, n = 51, Figure 6H), supporting a functional interaction between *twist* and *brahma*.

**Table 2:** Genetic interactions between *akirin* and BRM complex subunit genes.

Genotype	Embryos examined	% disrupted muscles^a^^,^ b
*twi^1^/+; akirin^2^/+*	60	21.6%
*twi^1^/+; akirin^3^/+*	105	18.1%
*akirin^2^/+,brm^I21^/+*	55	35%
*akirin^2^/+,moira^1^/+*	48	40%
*akirin^2^/+,Snr1^01319^/+*	52	45%
*Bap60^1^/+; akirin^3^/+*	109	20.2%
*akirin^2^/+,osa^308^/+*	52	42%
*akirin^2^,bap180^Δ86^*	45	38%
*twi^1/^+; brm^I21^/+*	51	23%

a Percentage of all stage 16 embryos examined that have missing, misattached, or duplicated muscles in 2 or more hemisegments.

b Crosses of *akirin* mutants with control balancer stocks (n>50) showed similar defects in >5% of stage 16 embryos examined.

To determine whether the Akirin protein associates with the BRM complex *in vivo*, we attempted co-immunoprecipitation experiments from embryonic lysates using tagged Akirin. While we did observe weak physical interaction between tagged Akirin and the Brahma core subunit (data not shown), these appear to be highly transient and not robust. As we had identified a functional interaction between *twist* and *brahma* (Figure 6H), we examined whether Brahma and Twist physically interact. Antibodies against Brahma successfully co-immunoprecipitated Twist from whole embryonic extracts (Figure S11). Together these data suggest that Twist and Akirin both interact functionally with the BRM complex during myogenesis.

### Twist, Akirin, and a core BRM complex subunit are localized to Twist-dependent enhancers *in vivo*

**Figure 7:**
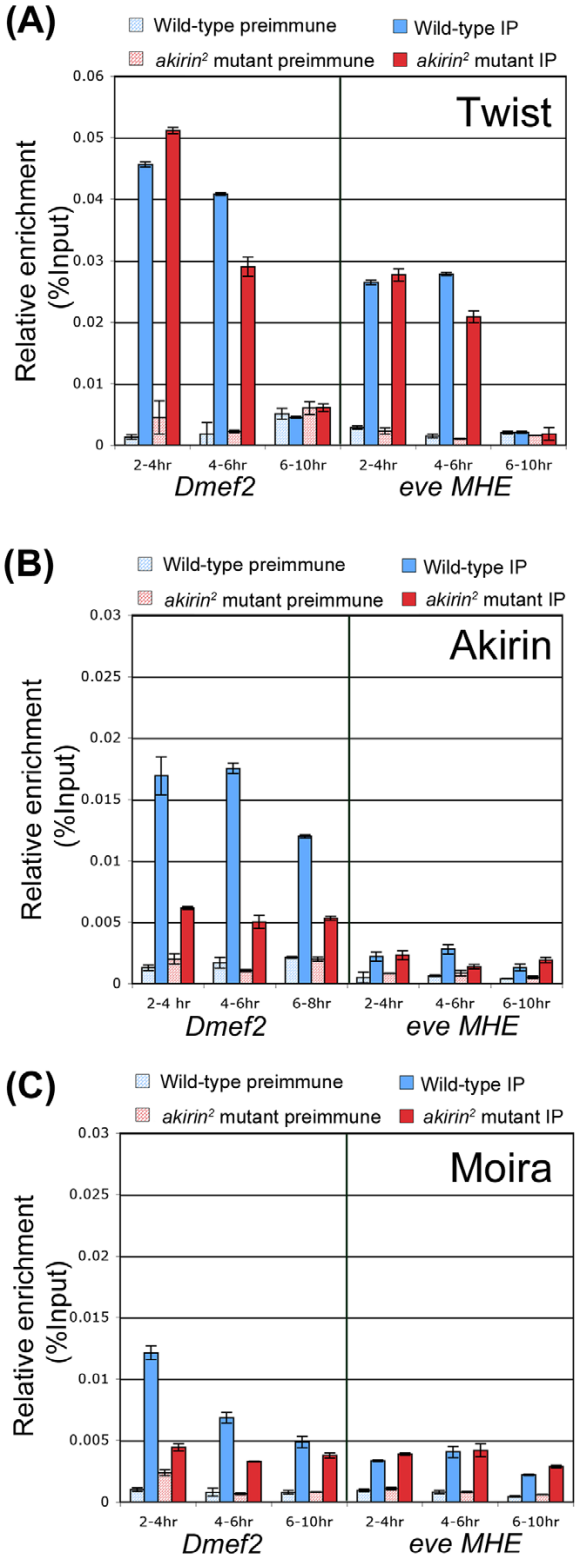
Akirin and the BRM complex co-occupy the *Dmef2* enhancer during embryogenesis. Chromatin immunoprecipitations were performed on extracts prepared from 2–4, 4–6, and 6–10 hour-old *yw* (Blue bars) and *akirin^2^* mutant (Red bars) embryos using anti-Twist (A), anti-Akirin (B), and anti-Moira (C) antibodies. The presence of the *Dmef2* enhancer or *even-skipped* MHE enhancer (*eve* MHE) was assayed in precipitated DNA samples using quantitative PCR. Values for preimmune (stippled bars) and immunoprecipated samples (solid color bars) are presented as percent of total input fraction. Akirin and the core BRM subunit Moira both co-occupy the *Dmef2* enhancer with Twist in 2–4 and 4–6 hour-old embryos. The occupancy of Moira at this enhancer appears to be Akirin-dependent, as Moira occupancy decreases by approximately 64% in *akirin* mutant extracts. (A–C) While Twist and Moira occupy the *eve* MHE enhancer, Akirin is not enriched at this region in wild-type or mutant extracts, and the absence of Akirin does not affect the occupancies of either Twist or Moira.

To further support our interaction data, we next tested whether Twist, Akirin, and the BRM complex were localized to Twist-regulated enhancer elements in embryos when the muscle pattern is being established. Using chromatin immunoprecipitation and quantitative PCR, we examined the occupancy of the *Dmef2* enhancer and the *eve* MHE element by Twist and Akirin (Figure 7). Both of these enhancer regions contain E-box elements that are bound by and regulated by Twist during development [8], [33], [46], [59]. In agreement with previously published data [8], antibodies against Twist and Akirin both successfully immunoprecipitated the *Dmef2* enhancer in extracts prepared from 2–4 and 4–6 hour-old embryos (Figure 7A, 7B). These data suggested that Akirin localizes to the *Dmef2* enhancer element with Twist during these time periods. However, while Twist occupancy at the *eve* MHE was detected as previously reported [8], Akirin protein was not significantly enriched at the *eve* MHE region in 2–4, 4–6, and 6–10 hour embryo extracts compared to preimmune antisera controls. We concurrently examined occupancy of these elements by Twist and Akirin in extracts prepared from *akirin^2^* mutant embryos. As we would predict, Twist occupancy at both *Dmef2* and *eve* MHE enhancers was not significantly affected by the absence of Akirin (Figure 7A).

We next tested the occupancy of *Dmef2* and *eve* MHE enhancers by a BRM subunit. We found that the BRM core subunit Moira occupied the *Dmef2* enhancer early, in extracts prepared from 2–4 hour old embryos (Figure 7C), with a similar occupancy profile as that observed with Twist at this time. However, the reduction in Akirin level appeared to affect Moira occupancy at the *Dmef2* enhancer, as we observed a 64% reduction in Moira levels at this enhancer in 2–4 hour old *akirin^2^* mutant extracts versus wild-type. We further found that Moira occupied the *eve* MHE, albeit at relatively low levels, at all time points examined. Further, this occupancy was not affected by the absence of Akirin, as the levels did not appear to change in *akirin^2^* mutant extracts versus wild-type (Figure 7C). For comparison, we also examined occupancy of Twist, Akirin and Moira at the enhancer region of the *oskar* gene, which is not normally expressed in the embryo at this time [60]. We found no detectable occupancy of any of these factors at this region at the same timepoints (see Figure S12). In summary, we find that Twist, Akirin, and core subunits of the BRM complex all co-occupy the *Dmef2* Twist-dependent enhancer together at a temporally critical time for establishing the muscle pattern during embryogenesis, and the occupancy of Moira at this element is dependent upon the presence of Akirin. In contrast, we find that Akirin is not present at a second Twist-regulated enhancer, the *eve* MHE, at the same period of embryonic development.

## Discussion

The Twist transcription factor controls many key processes in the establishment and patterning of the mesoderm, including organogenesis of the somatic body wall musculature during *Drosophila* embryonic development [6]. As Twist activity regulates a large number of genes and processes over the course of mesodermal development [8], [9], specific Twist functions are likely conferred through secondary and tertiary proteins that interact with Twist [11]. In a screen for such interacting proteins, we identified the highly conserved nuclear protein Akirin. *Akirin* mutants show defects in myogenesis consistent with a role in regulating Twist activity, in particular at the *Dmef2* gene. Akirin accomplishes this regulation through interactions with core subunits of the Brahma chromatin remodeling complex. Finally, we find Twist, Akirin and a BRM complex subunit all co-occupy the *Dmef2* enhancer during early embryogenesis, and this occupancy of a core BRM subunit requires the presence of Akirin. These data suggest that Akirin is required for optimal expression of this *Dmef2* enhancer element. By contrast, the Twist-regulated *eve* MHE regulatory element does not require Akirin for its expression. Together, these results suggest that Akirin is an accessory protein that links Twist and the BRM chromatin remodeling complex for activation of specific Twist-regulated enhancers during *Drosophila* embryonic development. We propose that this Akirin-mediated link between Twist and the Brahma complex represents a novel paradigm for providing tissue and target specificity for Twist transcription factor activity with chromatin remodeling machinery during embryogenesis.

### Akirin and Twist interact during embryonic development

Establishment of the somatic musculature during embryogenesis requires precisely regulated Twist activity [4]. The muscle phenotype of *akirin* mutants and *twist* and *akirin* double heterozygotes indicates a functional interaction between Twist and Akirin during myogenesis. This interaction is direct, as Akirin and Twist physically bind. Additionally, both Twist and Akirin co-occupy the *Dmef2* enhancer during embryogenesis, and their association is important for robust expression from the *Dmef2* enhancer. Dmef2 is a critical regulator of myogenesis and is expressed throughout this process [33], [40], [45], [47], [48]. Dmef2 coordinates multiple processes necessary for proper somatic myogenesis and regulates, in combination with Twist, a subset of Twist-regulated genes in a feed-forward manner [8], [47]. The missing, misattached, or duplicated muscle phenotypes that we observe in *akirin* mutants are similar to the phenotypes of embryos in which *Dmef2* or *twist* expression levels are modified [11], [40]–[42]. For example, missing and misattached muscles were reported in incompletely rescued *Dmef2* mutant embryos [41], while muscle duplications have been reported as a result of RNAi-mediated knockdown of *twist* [11]. Therefore, *akirin* mutant muscle phenotypes likely reflect a perturbation in Twist activity, resulting in an early alteration in *Dmef2* expression levels during embryogenesis. There are other elements in the *Dmef2* enhancer that are bound and controlled by transcription factors such as Pannier, Medea and Tinman, that regulate *Dmef2* expression during embryogenesis [8], [33], [46], [59]. It is unknown whether Akirin interacts with these factors at other *Dmef2* control elements for their optimal expression or whether interaction with just one factor is sufficient. These questions are subjects for further study.

Whole genome ChIP-on-chip experiments have identified almost 500 cis-regulatory elements that are bound by Twist during mesodermal development [8]. Further, Twist co-regulates a number of targets together with Dorsal, Dmef2 and Tinman in a feed-forward manner. Interestingly, our data would argue that Twist does not require Akirin for optimal expression of all Twist-dependent enhancers. First, our analysis of polytene chromosomes indicates that Akirin colocalizes with Twist at only 54% of Twist binding sites. Second, our chromatin immunoprecipitation experiments indicate that Akirin is enriched at the *Dmef2* enhancer during the first 10 hours of embryonic development, but not at the *even-skipped* MHE enhancer at the same time. Accordingly, we did not observe a noticeable decrease in Eve expression, a reduction in Eve-positive clusters, or defects in patterning of the DA1 muscle in *akirin* mutants (Figure 3, Figure S3, and data not shown). What could explain the different requirements for Akirin for optimal expression of *Dmef2* versus *eve*? On one level, there are inherent differences in the complexities of these enhancers: While both the *eve* MHE and *Dmef2* enhancers contain E-boxes bound and regulated by Twist, the *eve* MHE element also contains multiple DNA elements bound by a large array of factors from the Wingless, Decapentaplegic, and RTK-Ras-MAPK pathways [59]. This greater complexity of regulation may obviate the need for Akirin to achieve optimal expression. On another level, the difference in Akirin regulation of these enhancers could be reflected in the local chromatin environment of these enhancers, and whether the individual enhancer needs to be remodeled by SWI/SNF activity for optimal expression. Analysis of deposited Modencode data [61] suggests that the histone modification profile of the *eve* MHE remains largely static during the same temporal window as our chromatin immunoprecipitation experiment, while the environment surrounding the *Dmef2* enhancer element changes during this same time (data not shown). Further studies are required to identify the basis of this different requirement for Akirin at these Twist targets. Our polytene analysis indicates that a large number (>200) of Akirin-positive bands colocalize with Twist in the genome (Figure 4). Identifying the complete list of Twist target genes that require Akirin will likely yield further clues as to the regulatory logic of Akirin together with chromatin remodeling during Twist target expression.

### Akirin interactions with transcription factors other than Twist

Our data establishes Akirin as a Twist-interacting protein that promotes expression from a Twist-regulated enhancer; however, the results presented in this study also indicate that Akirin does not act solely with Twist.Analysis of salivary gland polytene chromosomes demonstrated that Akirin is associated with numerous actively transcribed gene loci. Twist is not normally expressed in salivary glands, therefore this result suggests that Akirin has roles in activation of non-Twist regulated genes. Moreover, the widespread expression of Akirin throughout the entire embryo suggests that specificity of Akirin function is determined not by restriction of Akirin expression, but rather by the associated transcription factor. Indeed, potential interactions between Akirin and other transcription factors have been described: Akirin misexpression enhances phenotypes resulting from mutations in the GATA-2 homologue *pannier* [62]. Additionally, whole genome yeast 2-hybrid analysis [57] suggests an interaction between Akirin and Charlatan, a zinc-finger transcription factor involved in development of the peripheral nervous system [63]. Finally, recent work has identified Akirin as a promyogenic factor and target for Myostatin regulation [30], as well as NF-κB target gene expression in the *Drosophila* innate immunity pathway [29]. Taken together, these interactions with transcription factors other than Twist, and roles in non-Twist-dependent pathways further support our model whereby Akirin functions as a general transcription cofactor. We propose that the regulatory mechanism involving Akirin and the Brahma chromatin remodeling complex at specific enhancers is applicable to these transcriptional regulators in these other contexts. Further experimentation is required to validate this model.

### Akirin is likely a novel accessory of the Brahma chromatin remodeling complex

Our studies identify Akirin as a nuclear factor that genetically interacts with the BRM complex and is required for optimal expression of the Twist-dependent *Dmef2* enhancer. This association between Akirin and BRM complexes is likely the mechanism whereby Akirin is linked to gene activation. The Brahma complex (BRM) promotes gene activation by remodeling the local chromatin environment allowing components of the general transcription machinery greater accessibility to the DNA [15]. BRM complexes are tightly associated with regions of transcriptionally active chromatin, and are associated with both promoter paused (initiating) and actively elongating RNA Polymerase II complexes throughout the *Drosophila* genome [23]. Although a strong physical association of BRM complexes with RNA Polymerase II has not been confirmed, loss of BRM function leads to a severe impairment in transcription by RNA polymerase II [23]. Based on our genetic and ChIP data, we conclude from our results that Akirin is not a core BRM subunit, but is rather an accessory protein that is capable of interacting with BRM complexes. We base this conclusion on the observation that the distribution of Akirin and BRM subunits on polytene chromosomes do not completely overlap, and because numerous biochemical analyses of BRM complex composition to date have failed to identify Akirin as a BRM subunit [24], [64]. Further, we did not observe a specific interaction of *akirin* with either BAP or PBAP complex-specific subunits during myogenesis (Figure 6). In *Drosophila*, both BAP and PBAP complexes have both been linked to gene activation and repression, are present in the same cells, and perform unique, yet cooperative, functions during development [15], [16], [19], [24]–[28]. Indeed, while we were able to observe weak physical interactions between Akirin and the Brahma core subunit *in vivo* (data not shown), these interactions were not overly robust. Moreover, it is unknown if Akirin needs to be post-translationally modified or to further associate with other factors to mediate a physical interaction with the Brahma subunit. Further, while we tested interactions with the core Brahma subunit, it remains to be determined whether Akirin may be interacting instead with other core BRM subunits. Nevertheless, our data strongly suggests a likely association as an accessory of the BRM complex. As an accessory protein, Akirin would likely confer tissue, target, and even temporal specificity on BRM complex activity by connecting BRM complexes with a particular transcription factor for promotion of gene expression (see below).

### Interactions among Akirin, BRM complexes, and Twist

Our data suggest that Twist target genes have different requirements for the presence of chromatin remodeling factors during gene activation and imply that the chromatin environments at these genes are varied. This also would suggest that the local chromatin environment of a particular Twist target changes over developmental time. Further experiments will be required to validate this hypothesis. As an accessory protein, Akirin optimizes Twist transcription factor activity outputs. Akirin likely accomplishes this optimization by facilitating an interaction between Twist and BRM complexes and as such, we would predict, change the local chromatin environment to one more favorable for transcription. The exact nature of the interface between bHLH transcription factors such as Twist and chromatin remodeling complexes such as BRM has not been previously determined. Our data would suggest that Akirin would be a suitable candidate for mediating this relationship between Twist and chromatin remodeling complexes. Mammalian SWI/SNF complexes are positively associated with bHLH transcription factor activity; however, the precise role of their remodeling activity during expression of bHLH target genes remains unclear [65]. Whether a similar linkage via Akirin is at play with mammalian Twist during development or in a cancer context remains to be tested. Nevertheless, in keeping with our proposed model of Akirin function, our data suggest a relationship between Twist and BRM during development: *twi^I1^/+;brm^2^/+* double heterozygous embryos show muscle patterning defects similar to *twi^1^/+;akirin^2^/+* double heterozygotes (Figure 6). Also, forced expression of Twist in salivary glands and subsequent analysis of colocalization on polytene chromosomes indicated that Twist and Brahma colocalized 58% of the time (Figure S11), a frequency similar to that observed between Twist and Akirin (54%, Figure 4 and data not shown).

Our finding that early (i.e., 2–4 hours) occupancy of the Moira core subunit at the *Dmef2* enhancer was decreased in *akirin* mutants would suggest that Akirin contributes to BRM complex localization. However, our co-immunoprecipitation experiments (data not shown) would suggest that any physical interaction between these two proteins would be either highly transient, or exquisitely sensitive to the presence of interfering factors such as protein tags. Therefore, the mechanism by which Akirin would increase Moira occupancy remains unclear. The result of such a recruitment or stabilization of BRM complexes by Akirin at Twist-target loci, would presumably result in remodeling of the local environment by BRM for optimal gene expression. Further experiments, aimed at understanding the nature of the Akirin/BRM complex association are currently underway. Together, this association between Twist, Akirin and the BRM complex would provide a novel mechanism linking chromatin remodeling factors to spatiotemporal-specific gene activation by the Twist transcription factor. Our work provides another venue to investigate how changes in the chromatin environment at specific targets leads to optimal gene expression and how these local changes impact the development of specific tissues.

## Materials and Methods

### *Drosophila* genetics

Flies were reared using standard conditions. Three different alleles of *akirin* were used: *akirin^KG01343^* (referred to in this work as *akirin^2^*), *akirin^EY08097^* (*akirin^1^*) and *akirin^EP(3)0906^* (*akirin^3^*). *akirin^1^* and *akirin^2^* are viable P-element insertions into the first intron of *akirin*, while the homozygous lethal *akirin^3^* allele is an insertion into the first exon (Figure 1A). We further generated two additional homozygous lethal *akirin* alleles by excision of the *akirin^1^* (*EY08097*) P-element (*akirin^4^* and *akirin^5^*). Mobilization of the *EY08097* P-element resulted in the isolation of two different mutations: *akirin^5^*, which is an insertion of approximately 1 kb of DNA into the second *akirin* exon due to incomplete P-element excision, and *akirin^4^*, which is a deletion of 6 kb containing the first exon and intron of *akirin*, as well as a portion of the upstream *SH3β* open reading frame. The *akirin^EP(3)0906^* mutant chromosome possessed a second unidentified lethal mutation in addition to the *EP(3)0906* insertion. This second lethal mutation was removed by recombination with a *rucuca*-marked chromosome [66] to produce the *akirin^3^* line used in this study. *akirin^3^*, *akirin^4^*, and *akirin^5^* homozygous mutant embryos die shortly after stage 16 and do not hatch into larvae (data not shown). For analysis, *akirin^3^*, *akirin^4^*, and *akirin^5^* were balanced over *TM3*, *Dfd-lacZ* or *TTG* [67]. For genetic interactions, the following alleles were used: *moira^1^*, *Snr1^01319^*, *brm^2^* and *brm^I21^* (Bloomington stock collection). *osa^308^* and *bap180^Δ86^* (a gift of J. Treisman), *Bap60^1^* (a gift of G. Mardon), *nurf^38^, Iswi^1^, and Su(var)3-9^1^* (a gift of V. Corces), and *twi^1^* [39]. *Dmef2-lacZ* [33] and *eveMHE-lacZ* (a gift of A. Michelson) were used for expression studies. GAL4 expression lines [68] used were *twi-GAL4;Dmef2-GAL4* [69] and *Sgs3-GAL4* [56]. *OreR* and *yw* flies were used as wild-type stocks where indicated. Germline clones [37] were generated by heat shock of *hs-FLP; ovoD^1^,FRT2A/akirin^3^,FRT2A* larvae. Germline clone females were then mated to *akirin^3^/TM3,Dfd-lacZ* at 20–22°C to create *akirin^3^* maternal/zygotic embryos.

### Production of UAS-HA–tagged Akirin flies

The HA-tag was fused in-frame at the Akirin C-terminus using PCR techniques (Table 3). PCR products were cloned into pUASt and injected into *yw* embryos as described [4]. Multiple independent transformant lines were generated and evaluated for Akirin-HA expression.

**Table 3:** Primers used in this study.

Sequence	Purpose
5′-CGCCTCGAGGCTTAGCCAGCGTAGTCTGGGACGTCGTATGGGTACGACAGGTAGCTAGGCGCTGC- 3′	Upstream primer for HA-tagging of Akirin
5′-CTCCCCCGTCTCCATAAAGGTC-3′	Upstream primer- production of *Dmef2* ISH probe
5′-GTTGCTACTGGTGCTGCTGCTG-3′	Downstream primer- production of *Dmef2* ISH probe
5′-AACTGCCAAGCGTGTGCCGTGT-3′	qPCR detection of *Dmef2* transcript- upstream primer
5′-CAAGGCCAAAGGGGCAGCACCA-3	qPCR detection of *Dmef2* transcript- downstream primer
5′-CTTCTTCAGCGACACCCATT-3′	qPCR detection of *GAPDH* transcript- upstream primer
5′-ACCGAACTCGTTGTCGTACC-3′	qPCR detection of *GAPDH* transcript- downstream primer
5′-CCGATGCTGCTGCTGCTGCTACT-3′	Forward qPCR primer used in ChIP of *Dmef2* enhancer (-2344)
5′-GACCATGTACCCCGATGCTGTGC-3′	Reverse qPCR primer used in ChIP of *Dmef2* enhancer (-2257)
5′- CTCCCCCGTCTCCATAAAGGTC-3′	Forward qPCR primer used in ChIP of Dmef2 ORF (+11007)
5′- GTTGCTACTGGTGCTGCTGCTG-3′	Reverse qPCR primer used in ChIP of Dmef2 ORF (+10782)
5′- GGAAATCGTCTTGGGATGCGAGTGGT-3′	Forward qPCR primer used in ChIP of *eve* MHE region (+6171)
5′- AGCTGCAGATCCGGACTCGCAATAG-3′	Reverse qPCR primer used in *eve* MHE region (+6334)
5′- CTGGGTCGCTTGGAGAAGGAGTT-3′	Forward qPCR primer used in ChIP of *eve* ORF (+490)
5′- CGATCCTCTGACGCTTGTCCTTC-3′	Reverse qPCR primer used in eve ORF (+616)
5′-TGCGAATGGTCTTCATGGAA-3′	Forward qPCR primer used in ChIP of *oskar* enhancer (+8117)
5′-CACCGTCAAGCAGCGTGTAC-3′	Reverse qPCR primer used in ChIP of *oskar* enhancer (+8182)

### Double-interaction screen

Briefly, we adapted the method described by L. Pick and colleagues [31]. Enhancers tested were the 175-bp upstream enhancer of *Dmef2* [33] and the 375-bp enhancer contained in the first intron of the *tinman* gene [34] cloned upstream of a *HIS3* reporter. Reporter strains were created using these constructs. Screening for Twist-interacting proteins was performed by introducing a cDNA library prepared from 0–6 hour *Drosophila* embryos fused to the GAL4 activation domain [31]. Akirin was identified as a cDNA that expressed the *HIS3* reporter in a Twist-dependent manner at both *Dmef2* and *tinman* enhancers [32].

### Immunohistochemistry and production of Akirin antibody

Antibodies and dilutions used: anti-tropomyosin (1∶1000, Abcam), anti-myosin monoclonal antibody (1∶500, a gift of S. Abmayr), anti-Twist (1∶100, a gift of M.Levine) anti-Osa (1∶50, DSHB), anti-Brm (1∶50, gift of C.P. Verrijzer), anti-HA 3F10 (1∶25, Roche), anti-Dmef2 (1∶1000, a gift of B. Patterson), anti-Snr1 (1∶100, gift of A. Dingwall), anti-Eve (1∶3000, a gift of M. Frasch), anti-phosphohistone H3 (mouse monoclonal, 1∶100, Millipore), anti-Serine-7-phosphorylated RNA polymerase II (1∶100, Millipore) and anti-β-galactosidase (1∶1000, Abcam). Whole-mount embryo immunohistochemistry was performed as described [70]. Comparison of β-galactosidase expression levels in wild-type and *akirin* mutant embryos was performed as per [11]. For production of polyclonal Akirin antibody, the peptide sequence of CESMIKERENQLR corresponding to residues 151–163 in full-length *Drosophila* Akirin was synthesized and injected into rabbits (Sigma). Production bleeds were tested for immunoreactivity using pre-immune serum as a negative control (See Figure S2).

### Microscopy

Bright field and immunofluorescent images were obtained using a Zeiss Axiophot microscope. Images were processed using Adobe Photoshop. Confocal images were acquired using either a Zeiss LSM 510 confocal scanning system mounted on an Axiovert 100 M microscope equipped with a 63× 1.2 NA C-Apochromat water objective, or a Leica SP5 confocal microscope equipped with a 63× 1.4 NA HCX PL Apochromat oil objective. Pinholes were set to capture optical slices of 1.0 µm. All images were processed using Adobe Photoshop. Maximum intensity projections of confocal Z-stacks were rendered using Volocity Visualization (Improvision).

### GST-pulldown assays

Twist was cloned into pGEX-2T in frame and fusion protein expressed via IPTG induction. Glutathione Sepharose 4B beads (Amersham Pharmacia Biotech) bound with GST or GST-Twist were suspended in ZTx buffer (25 mM HEPES, pH = 7.5, 12.5 mM MgCl_2_, 20% glycerol, 0.1% Triton-X 100, 150 mM KCl)+0.25 mg/ml BSA and 1 mM DTT and incubated for 10 mins with rotation. ^35^S-Methionine-labeled Akirin and Daughterless proteins were produced *in vitro* using the TnT coupled reticulocyte lysate system (Promega). Beads and translated proteins were then incubated at room temperature with rotation for 1 hour. Beads were spun and washed in NETTx buffer (20 mM Tris, 100 mM NaCl, 1 mM EDTA and 0.25% Triton-X 100). Proteins were eluted from beads by boiling in Laemmli sample buffer and resolved by SDS-PAGE. Gels were fixed in 50% Methanol, 10% Acetic Acid, incubated in Amplify solution (Amersham Pharmacia Biotech), and dried for autoradiography.

### Western blotting

Whole-embryo extracts were prepared and resolved by SDS-PAGE as described [71]. Western detection was performed as described [72]. Antibodies and dilutions used: anti-Dmef2 (rabbit, 1∶1000) (a gift of B. Patterson), anti-alpha-tubulin (mouse, 1∶5000) (Sigma), anti-Brahma (1∶1000) (a gift of C.P.Verrijzer), anti-β-galactosidase (mouse, 1∶1000) (Promega).

### Densitometry

Autoradiographs were scanned using a BioRad GS-800 Calibrated Densitometer to determine optical density of each band, which was then divided by the area examined. For analysis of β-galactosidase levels (see Figure S5), this value was normalized to similar results obtained from anti-α-tubulin loading controls. For analysis of co-immunoprecipitations (see Figure 6), this value was reported as a percentage of the input sample analyzed. For each condition, background (chosen from blank region of the film piece, with no sample present) was subtracted from all analyzed regions prior to analysis.

### Polytene chromosome immunohistochemistry, in situ hybridization, and immunocolocalization analysis

Polytene squash preparations and immunostaining was performed as described [52]. For colocalization analysis, separate channel information was compared for signal bands in polytene squashes. Contrast settings were set to maximum for each channel, eliminating >80% of the background signal. A band was scored as “colocalized” when it appeared in both channels. Bands that appeared solely in either signal channel were scored accordingly. A minimum of 150 bands were counted from at least three different squash preparations. Data was plotted in percentage bar graphs using Microsoft Excel.

In situ probes were generated using the Roche DIG-labeled PCR kit, using oligonucleotides to amplify exon 8 of the Dmef2 open reading frame (Table 3). *In situ* probe purification and hybridization was performed as described [73]. Following hybridization, slides were washed in increasing stringency SSC solutions, and then incubated in polytene buffer. Antibody staining was carried out as described above, using 1∶100 rhodamine-conjugated anti-DIG antibodies (Roche) to detect DIG-labeled probes.

### Production of cDNA and quantitative PCR

wild-type (*yw*) and *akirin^2^* embryos were collected on 10 cm apple juice agar plates and allowed to grow to 4–6 hours of age after egg laying. Embryos were dechorionated in bleach and homogenized in RNA TriReagent (Sigma). Total mRNA was prepared according to manufacturer's instructions, and resuspended in RNAse-free water. Following purification, total mRNA was treated with DNAseI to degrade genomic DNA, and re-purified using phenol∶chloroform. 1 microgram of total mRNA was used to prepare first-strand cDNA using the RevertAid First Strand Synthesis Kit (Fermentas). First-strand product was diluted to produce a working concentration of 10 ng/µL. For quantitative PCR, first-strand cDNA product was diluted 1∶2 with Lightcycler 480 SYBR green master mix (Roche). qPCR was conducted using a Lightcycler 480 qPCR machine (Table 3). qPCR results were analyzed and relative ratios of *Dmef2* transcripts (normalized to *GAPDH* levels) in *yw* and *akirin^2^* mutants were determined using described methods [74].

### Co-immunoprecipitations

Total nondenatured extracts were prepared from *yw* embryos collected on apple juice plates. To prepare total nondenatured extracts, embryos were dechorinated in 50% bleach, rinsed in distilled water, and immediately homogenized in one mL of extraction buffer (50 mM HEPES, pH 7.6, 385 mM NaCL, 0.1% Tween-20, 0.1 mM EGTA, 1.1 mM MgCl_2_ and 100 µg/mL PMSF, and 1 µg/mL each of Aprotinin, leupeptin, and pepstatin A), using a 1 mL Dounce homogenizer. Extracts were spun at 15,000 g at 4°C to pellet debris. Supernatants were removed and following the addition of glycerol to 10%, extracts were snap-frozen in liquid nitrogen before storing at −80°C for later use. For immunoprecipitations, extracts were thawed and quantitated using the BCA assay method (Pierce). Extracts were diluted in IP buffer (10 mM HEPES pH 8, 100 mM NaCl, 10% glycerol, 0.05% Tween-20, 100 µg/mL PMSF, and 1 µg/mL each of Aprotinin, leupeptin, and pepstatin A) to produce a final concentration of 3 mg/mL of total protein. Extracts were incubated with 10 µL of anti-Brahma antibody (a gift of P. Verrijzer) for 1 hour at 4°C. Immunocomplexes were pulled-down using 20 µL of a 50% protein A-agarose slurry. Beads were washed four times at 4°C, and immunocomplexes were released by boiling in Laemmli sample buffer.

### Chromatin immunoprecipitation (ChIP)

Chromatin immunoprecipitations were performed essentially as described [75] with several modifications. In brief, 2–4 hour, 4–6 hour, 6–10 hour *yw Drosophila* embryos were collected, dechorionated, and fixed in fixing solution (1 mM EDTA, 0.5 mM EGTA, 100 mM NaCl, 2% formaldehyde (v/v), 50 mM HEPES, pH 8.0) with an equal volume of n-heptane by vigorous shaking for 25 min at room temperature. Fixed embryos were washed twice with methanol, once with storage buffer (50 mM Tris-HCl pH 8.0, 1 mM EDTA), and kept at −80°C. Approximately 100 µL of fixed embryos were washed with 1 ml of IP buffer (100 mM NaCl, 67 mM Tris pH 8.0, 0.33% SDS, 1.66% Triton X-100, 5 mM EDTA) for 10 min. The following procedures were all carried out at 4°C. Embryos were resuspended in IP buffer containing protease inhibitor cocktail, were then sonicated fourteen times for 12 sec by a Branson 250 sonicator at a power setting of 4 and 30% duty cycle. After sonication, the Whole Cell extract; (WCE) was obtained by centrifugation. 30 µL of WCE was diluted, reverse cross-linked, and treated with proteinase K. DNA was purified using QIAprep Spin Miniprep columns and recovered in 100 µL of elution buffer (Qiagen) to check genomic DNA fragments with an approximate bulk size of 300–800 bp. For each immunoprecipitation, WCE containing 75 µg chromatin DNA was treated with either 5 µL of nonimmune guinea pig serum, anti-Twist antiserum (guinea pig), anti-Akirin antiserum (rabbit), and anti-Moira antiserum (rabbit), together with 25 µL of protein A agarose coated with salmon sperm DNA (Upstate) for overnight incubation. Mock immunoprecipitations were performed using nonimmune guinea pig serum, preimmune rabbit serum, and nonimmune rabbit serum. Precipitated agarose beads were washed with mixed micelle buffer, buffer 500, LiCl/Detergent buffer, and TE, then treated with RNase A. The precipitated immune complexes were eluted by Bicarbonate/SDS buffer and cross-links were reversed in the presence of proteinase K overnight at 65°C. DNA was purified using QIAprep Spin Miniprep columns and recovered in 200 µL of elution buffer. Quantitative PCR was performed using the Applied Biosystems (ABI) Prism 7700 Real-Time qPCR instrument (AQ method). One of triplicates of reaction mixture were prepared by adding 4 ul 2× SYBR mix (usb 75762), 0.4 µL 2.5 uM Primers (Table 3), and 3.6 µL DNA template. To depict standard curves, duplicates of each 5, 50, and 500 folds diluted DNA from WCE as described above were used. %input and SD were calculated from triplicated scores of immunoprecipitations over that of input WCE.

## Supporting Information

Figure S1 Specificity of Akirin antibody. (A) Pre-immune serum and anti-Akirin immunoserum were used to stain duplicate Western blots containing wild-type whole-embryo extracts. Volume of extract loaded is indicated above in microliters. Anti-Akirin immunosera detects a double band of approximately 22 and 20 kDa. The same doublet is not detected with pre-immune serum at identical concentration. Anti-α-tubulin included as a loading control. (B) Akirin is significantly decreased in akirin^3^ mutant extracts. Twenty-five (25) akirin^3^ homozygous mutant and akirin^3^/+ 6–10 hr-old heterozygote embryos were loaded in each lane, and stained with anti-Akirin antibodies. (C) Polytene chromosomes were prepared from OregonR larvae and stained with preimmune serum or anti-Akirin immunoserum as indicated, at identical concentrations. Images were acquired with identical gain and acquisition settings. Scale bar = 5 µm.
(TIF)

Figure S2 Akirin protein is expressed broadly throughout the embryo and is localized to the nucleus. (A) Hemisegment of wild-type embryo immunostained with antibodies against Akirin and Lamin, which labels the nuclear envelope. Scale bar = 20 µm. (B) twi-GAL4;Dmef2-GAL4>UAS-Akirin-HA embryos stained with antibodies against HA and Twist. HA-tagged Akirin localizes to the nucleus with Twist in these embryos. Scale bar = 20 µm.
(TIF)

Figure S3 akirin mutant embryos display a range of mutant muscle phenotypes. (A) Genomic map of akirin locus, showing location of P-element insertions and corresponding akirin mutant alleles used in this study. (B) Whole embryo presentations of akirin mutant muscle phenotypes. Lateral views of stage 16 wild-type (i, wt) and akirin mutant (ii, iii, iv) embryos demonstrate the types of muscle phenotypes observed. All embryos stained with anti-tropomyosin to reveal somatic musculature. All allelic combinations are listed as maternal/paternal contribution. For clarity, the LT muscles are used to illustrate the following predominant muscle defects observed in akirin mutants (red arrows): (ii) improperly attached muscles, (iii) duplicated muscles, and (iv) missing muscles. In all figures, anterior is to left and dorsal is up. Scale bar = 50 µm. (C) akirin mRNA is maternally loaded. RT-PCR for akirin and twist mRNA performed using total mRNA isolated from 0–1 hour wild type embryos. Plasmid controls provided as positive amplification controls. rp49 amplification included as positive control for a maternally deposited message.
(TIF)

Figure S4 Founder cell markers appear unaffected in akirin mutant embryos. Wild-type (wt) or akirin mutant embryos (allelic combinations as indicated) were immunostained using antibodies against Even-skipped (stage 11 embryos, panels A–C), Krüppel (late stage 12, panels D–F) and Slouch (late stage 12, panels G–I). Scale bar = 50 µm.
(TIF)

Figure S5 Comparison of eveMHE-lacZ expression levels in wild-type and akirin mutant embryos. (A) Western blot of whole cell extracts prepared from transgenic wild-type and akirin^2^ mutant embryos carrying the Dmef2-lacZ reporter. Anti-α-tubulin staining provided as loading control. Densitometric analysis indicates that β-galactosidase expression levels are slightly reduced when normalized against tubulin controls (0.6 in wild-type versus 0.4 in akirin^2^ mutants). (B,C) Wild type (B) and akirin^2^ mutant (C) embryos carrying a lacZ transgene under the control of the even-skipped MHE element were stained with antibodies against β-galactosidase. Close-up of two hemisegments presented for comparison. No obvious difference in β-galactosidase expression was detected in akirin mutants. Scale bar in (B,C) = 20 µm.
(TIF)

Figure S6 Whole-genomic distribution of Akirin and active transcription markers in polytene chromosomes. Shown are the whole chromosome spreads that are referenced in Figure 4. Scale bar = 5 µm.
(TIF)

Figure S7 Ectopic overexpression of Twist in 3rd instar salivary glands induces expression of Dmef2. Twist was expressed in salivary glands using the Sgs3-GAL4 driver line. Expression of Dmef2 verified by Western blotting, anti-a-tubulin provided as loading control.
(TIF)

Figure S8 Colocalization of endogenous Akirin protein and expressed Akirin-HA. UAS-Akirin-HA was expressed in larval salivary glands using the Sgs3-GAL4 driver. Polytene chromosomes were prepared and immunostained with antibodies against endogenous Akirin (green) and HA (red). Representative regions of polytene squashes presented. Near-complete colocalization of endogenous Akirin and expressed Akirin-HA was observed (examples shown with white arrows).
(TIF)

Figure S9 Whole-genomic distribution of Akirin and (A) Brahma, (B) Snr1, and (C) Osa in polytene chromosomes. Shown are the whole chromosome spreads referenced in Figure 5. Scale bar = 5 µm.
(TIF)

Figure S10 Heterozygous embryos for BRM complex subunit members do not show muscle phenotypes. Stage 16 heterozygote embryos for indicated BRM complex subunit used in Figure 6 were stained with anti-myosin antibodies to show the body wall musculature. Heterozygous embryos were verified by immunostaining for marked balancers; balancer staining channel omitted for clarity. No body wall muscle phenotypes were observed in BRM complex subunit heterozygotes.
(TIF)

Figure S11 Twist and Brahma colocalize in the genome and physically interact. (A) Twist was expressed in salivary glands using the Sgs3-GAL4 driver line. Polytene chromosomes were prepared from Twist-expressing salivary glands and immunostained with antibodies against Twist (red) and Brahma (green). Polytene squashes were costained with DAPI to visualize the DNA. Colocalization between Twist and Brahma (select examples shown in white arrows) was observed in approx. 58% of gene loci. Scale bar = 5 µm. Background staining in green channel indicated with (*). (B) Antibodies against the Brahma core subunit successfully immunoprecipitated Twist from wild-type (yw) embryonic extracts. “Input” lanes correspond to 2.5% of total embryonic extract. Note that (B) is a composite figure; lanes were omitted from the same gel for sake of clarity and direct comparison.
(TIF)

Figure S12 Specificity of antibodies used in chromatin immunoprecipitation assays. Chromatin immunoprecipitations using antibodies against Twist, Akirin and Moira (dark blue) and preimmune antisera (light blue, see Figure 7) were examined for enrichment of the oskar enhancer. No enrichment of the oskar enhancer was detected in immunoprecipitations. To enable direct comparsion, vertical axis in (A), (B), and (C) is identical to corresponding charts in Figure 7. For example, at 2–4 hours, Twist enrichment at the Dmef2 versus oskar enhancers was 0.046 versus 0.0011. For Akirin, occupancy was 0.017 at the Dmef2 enhancer versus 0.00092 at oskar. For Moira, occupancy was 0.012 at the Dmef2 enhancer versus 0.00085 at oskar.
(TIF)
