# Supplementary figures and images for: Akirin Links Twist-Regulated Transcription with the Brahma Chromatin Remodeling Complex during Embryogenesis
Source: PLoS Genet. 2012 Mar 1;8(3):e1002547. doi: 10.1371/journal.pgen.1002547 (PMC3291577; doi:10.1371/journal.pgen.1002547)

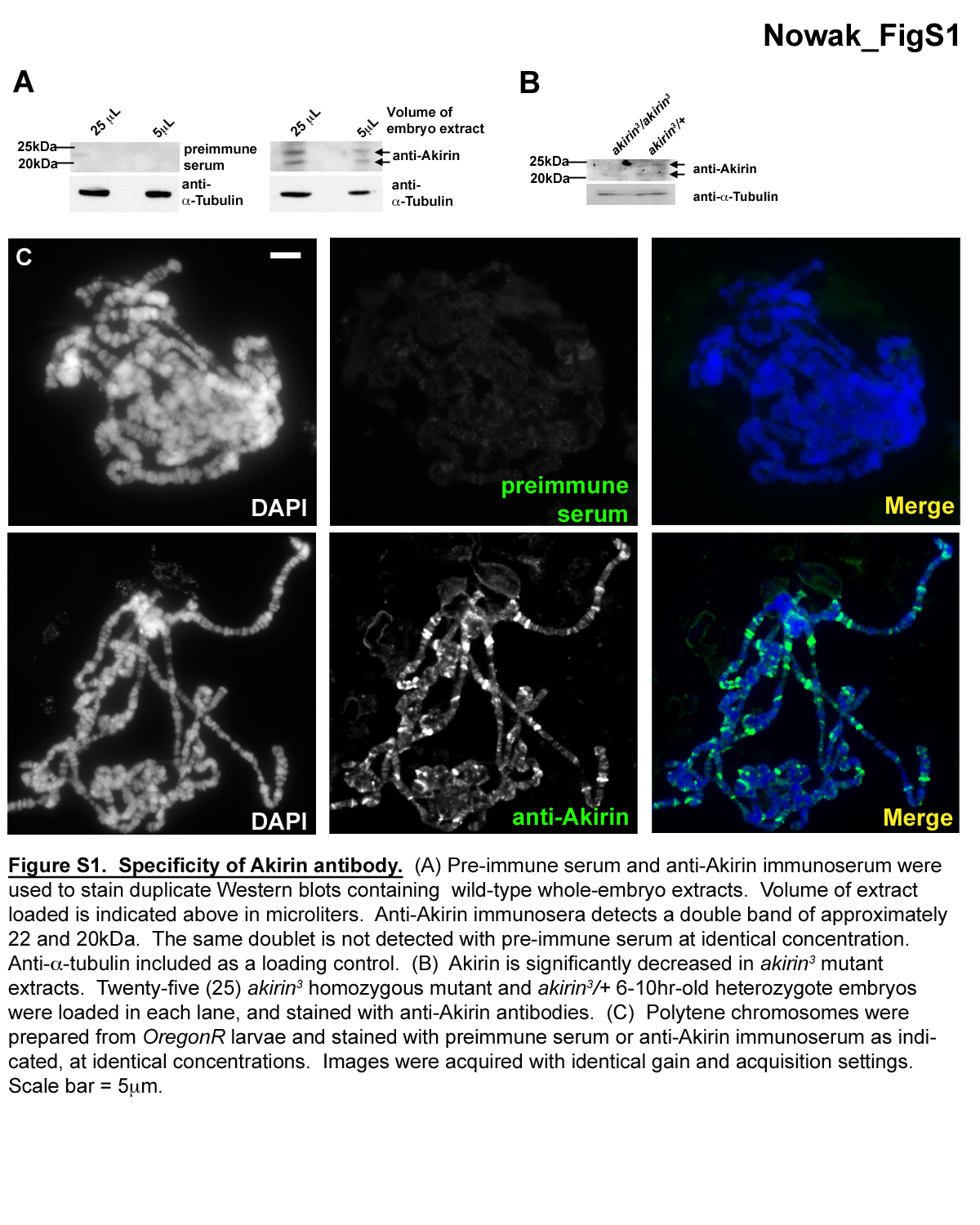

Supplement: Figure S1 — Specificity of Akirin antibody. (A) Pre-immune serum and anti-Akirin immunoserum were used to stain duplicate Western blots containing wild-type whole-embryo extracts. Volume of extract loaded is indicated above in microliters. Anti-Akirin immunosera detects a double band of approximately 22 and 20 kDa. The same doublet is not detected with pre-immune serum at identical concentration. Anti-α-tubulin included as a loading control. (B) Akirin is significantly decreased in akirin3 mutant extracts. Twenty-five (25) akirin3 homozygous mutant and akirin3/+ 6–10 hr-old heterozygote embryos were loaded in each lane, and stained with anti-Akirin antibodies. (C) Polytene chromosomes were prepared from OregonR larvae and stained with preimmune serum or anti-Akirin immunoserum as indicated, at identical concentrations. Images were acquired with identical gain and acquisition settings. Scale bar = 5 µm. (TIF) [file pgen.1002547.s001.tif]

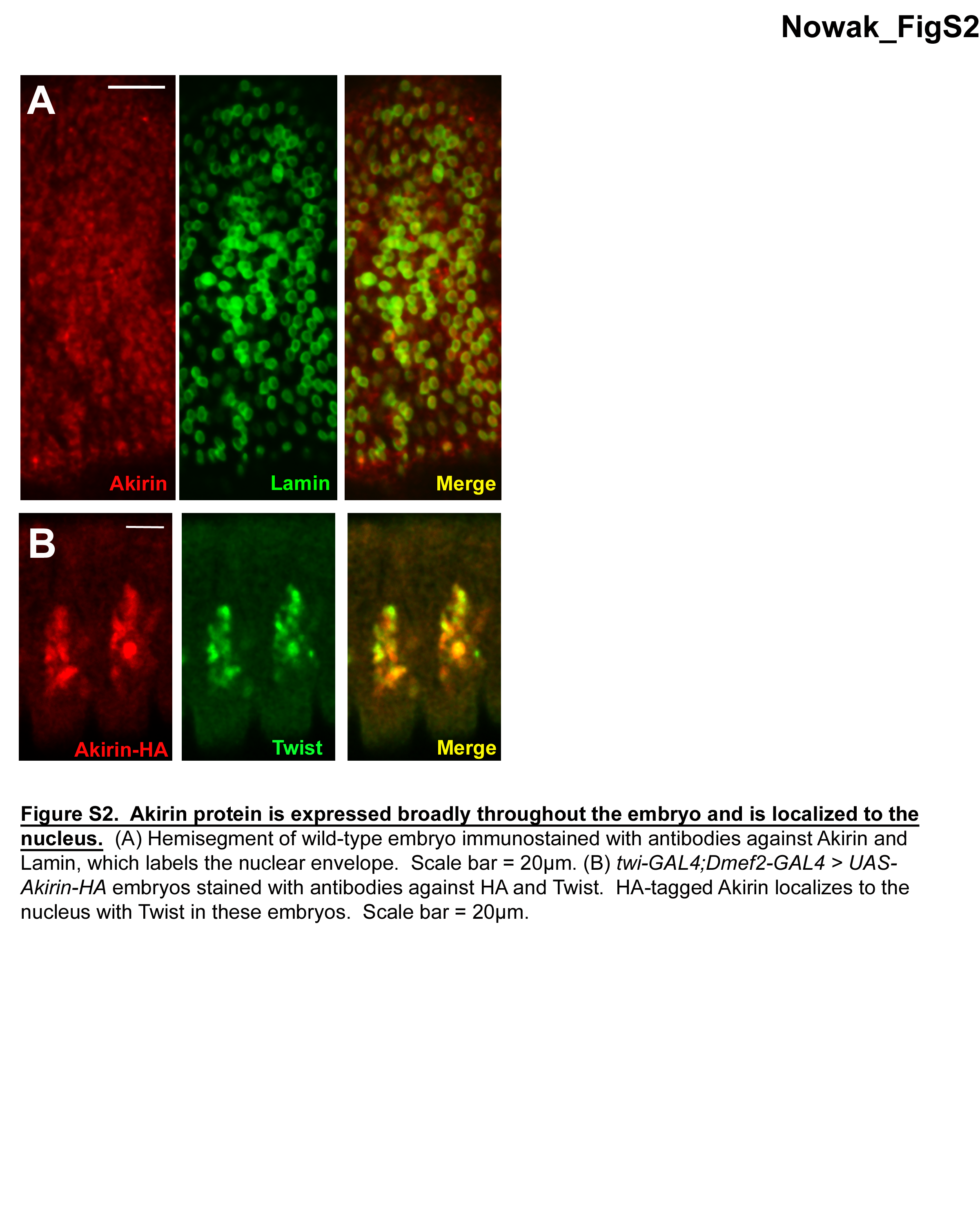

Supplement: Figure S2 — Akirin protein is expressed broadly throughout the embryo and is localized to the nucleus. (A) Hemisegment of wild-type embryo immunostained with antibodies against Akirin and Lamin, which labels the nuclear envelope. Scale bar = 20 µm. (B) twi-GAL4;Dmef2-GAL4>UAS-Akirin-HA embryos stained with antibodies against HA and Twist. HA-tagged Akirin localizes to the nucleus with Twist in these embryos. Scale bar = 20 µm. (TIF) [file pgen.1002547.s002.tif]

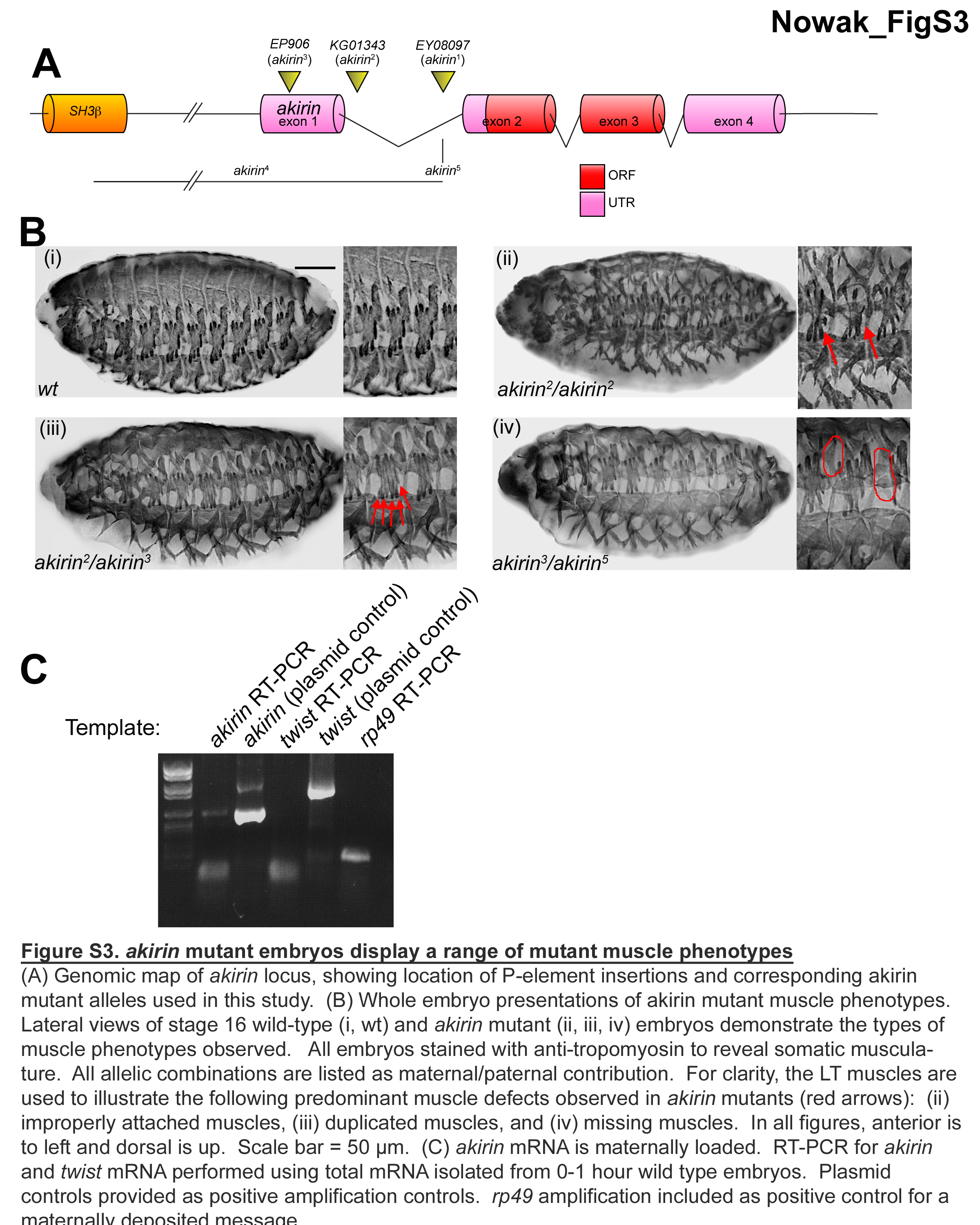

Supplement: Figure S3 — akirin mutant embryos display a range of mutant muscle phenotypes. (A) Genomic map of akirin locus, showing location of P-element insertions and corresponding akirin mutant alleles used in this study. (B) Whole embryo presentations of akirin mutant muscle phenotypes. Lateral views of stage 16 wild-type (i, wt) and akirin mutant (ii, iii, iv) embryos demonstrate the types of muscle phenotypes observed. All embryos stained with anti-tropomyosin to reveal somatic musculature. All allelic combinations are listed as maternal/paternal contribution. For clarity, the LT muscles are used to illustrate the following predominant muscle defects observed in akirin mutants (red arrows): (ii) improperly attached muscles, (iii) duplicated muscles, and (iv) missing muscles. In all figures, anterior is to left and dorsal is up. Scale bar = 50 µm. (C) akirin mRNA is maternally loaded. RT-PCR for akirin and twist mRNA performed using total mRNA isolated from 0–1 hour wild type embryos. Plasmid controls provided as positive amplification controls. rp49 amplification included as positive control for a maternally deposited message. (TIF) [file pgen.1002547.s003.tif]

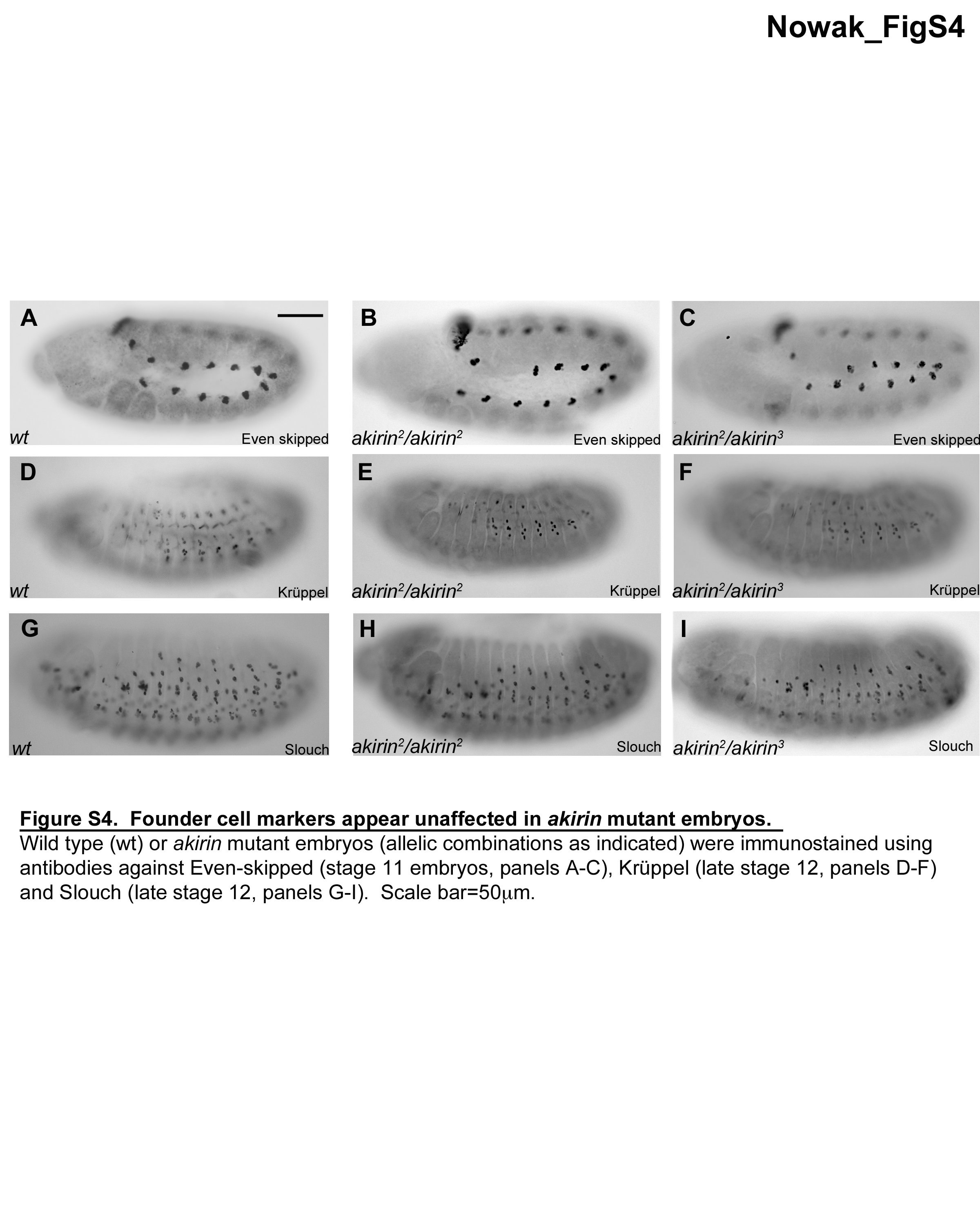

Supplement: Figure S4 — Founder cell markers appear unaffected in akirin mutant embryos. Wild-type (wt) or akirin mutant embryos (allelic combinations as indicated) were immunostained using antibodies against Even-skipped (stage 11 embryos, panels A–C), Krüppel (late stage 12, panels D–F) and Slouch (late stage 12, panels G–I). Scale bar = 50 µm. (TIF) [file pgen.1002547.s004.tif]

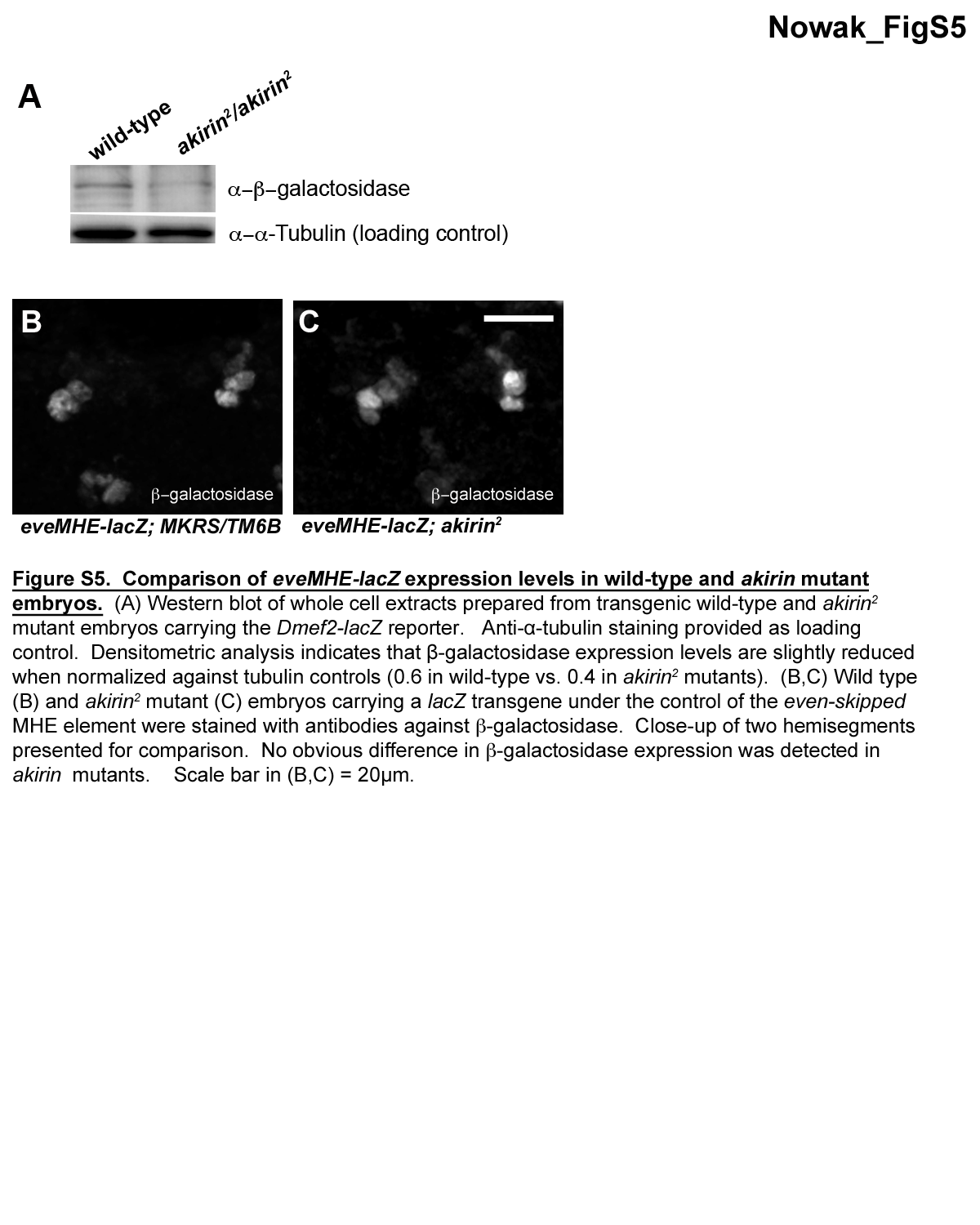

Supplement: Figure S5 — Comparison of eveMHE-lacZ expression levels in wild-type and akirin mutant embryos. (A) Western blot of whole cell extracts prepared from transgenic wild-type and akirin2 mutant embryos carrying the Dmef2-lacZ reporter. Anti-α-tubulin staining provided as loading control. Densitometric analysis indicates that β-galactosidase expression levels are slightly reduced when normalized against tubulin controls (0.6 in wild-type versus 0.4 in akirin2 mutants). (B,C) Wild type (B) and akirin2 mutant (C) embryos carrying a lacZ transgene under the control of the even-skipped MHE element were stained with antibodies against β-galactosidase. Close-up of two hemisegments presented for comparison. No obvious difference in β-galactosidase expression was detected in akirin mutants. Scale bar in (B,C) = 20 µm. (TIF) [file pgen.1002547.s005.tif]

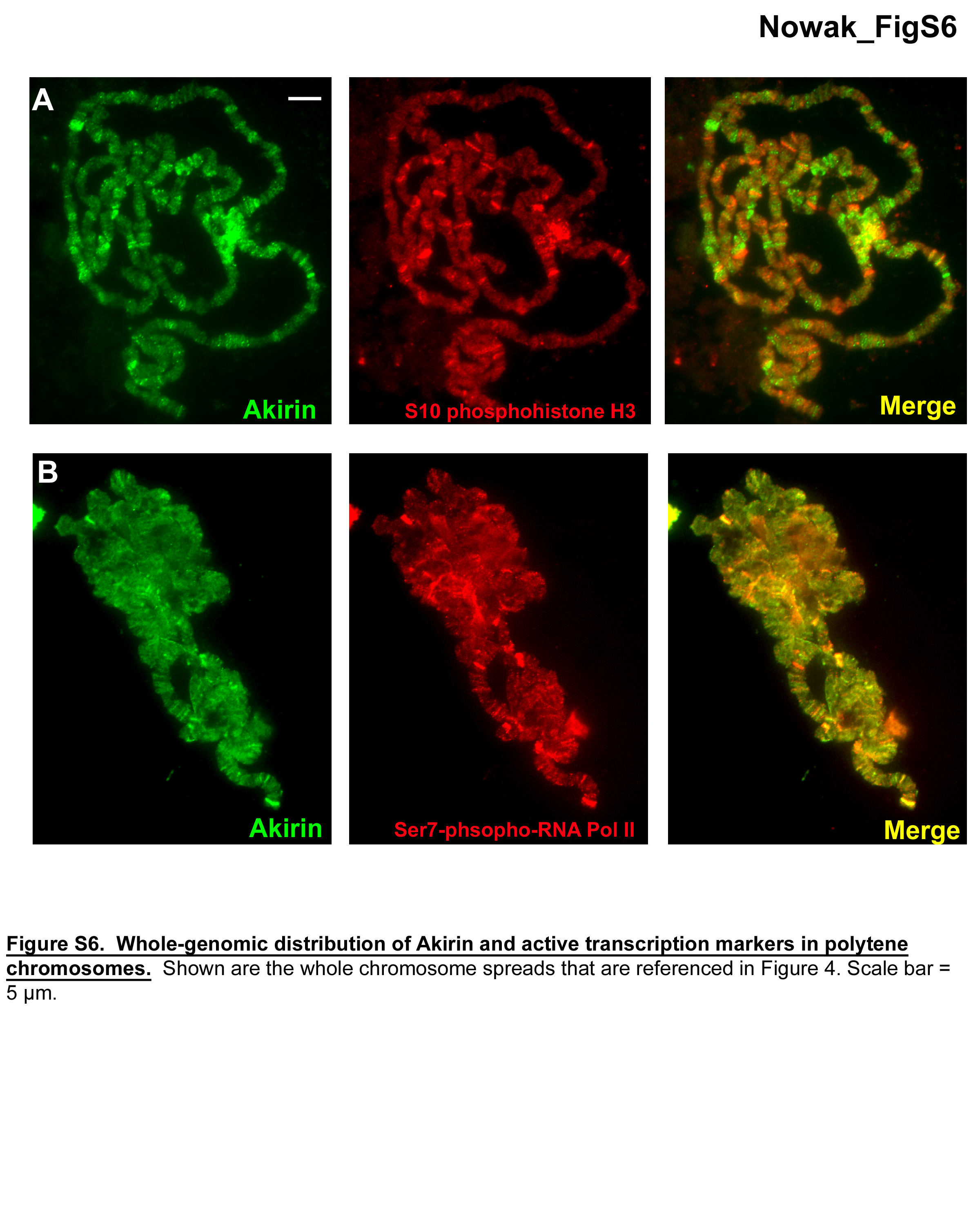

Supplement: Figure S6 — Whole-genomic distribution of Akirin and active transcription markers in polytene chromosomes. Shown are the whole chromosome spreads that are referenced in Figure 4. Scale bar = 5 µm. (TIF) [file pgen.1002547.s006.tif]

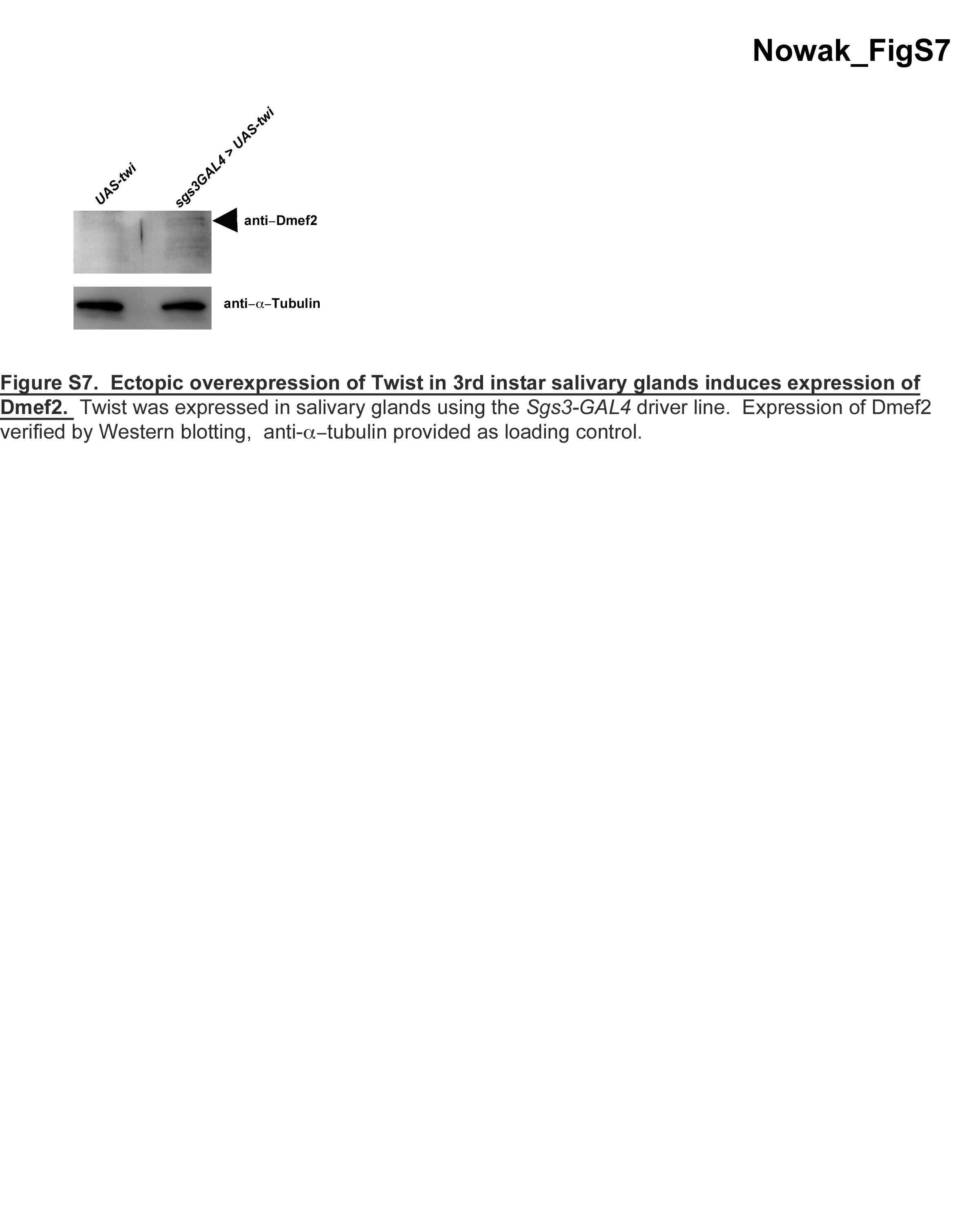

Supplement: Figure S7 — Ectopic overexpression of Twist in 3rd instar salivary glands induces expression of Dmef2. Twist was expressed in salivary glands using the Sgs3-GAL4 driver line. Expression of Dmef2 verified by Western blotting, anti-a-tubulin provided as loading control. (TIF) [file pgen.1002547.s007.tif]

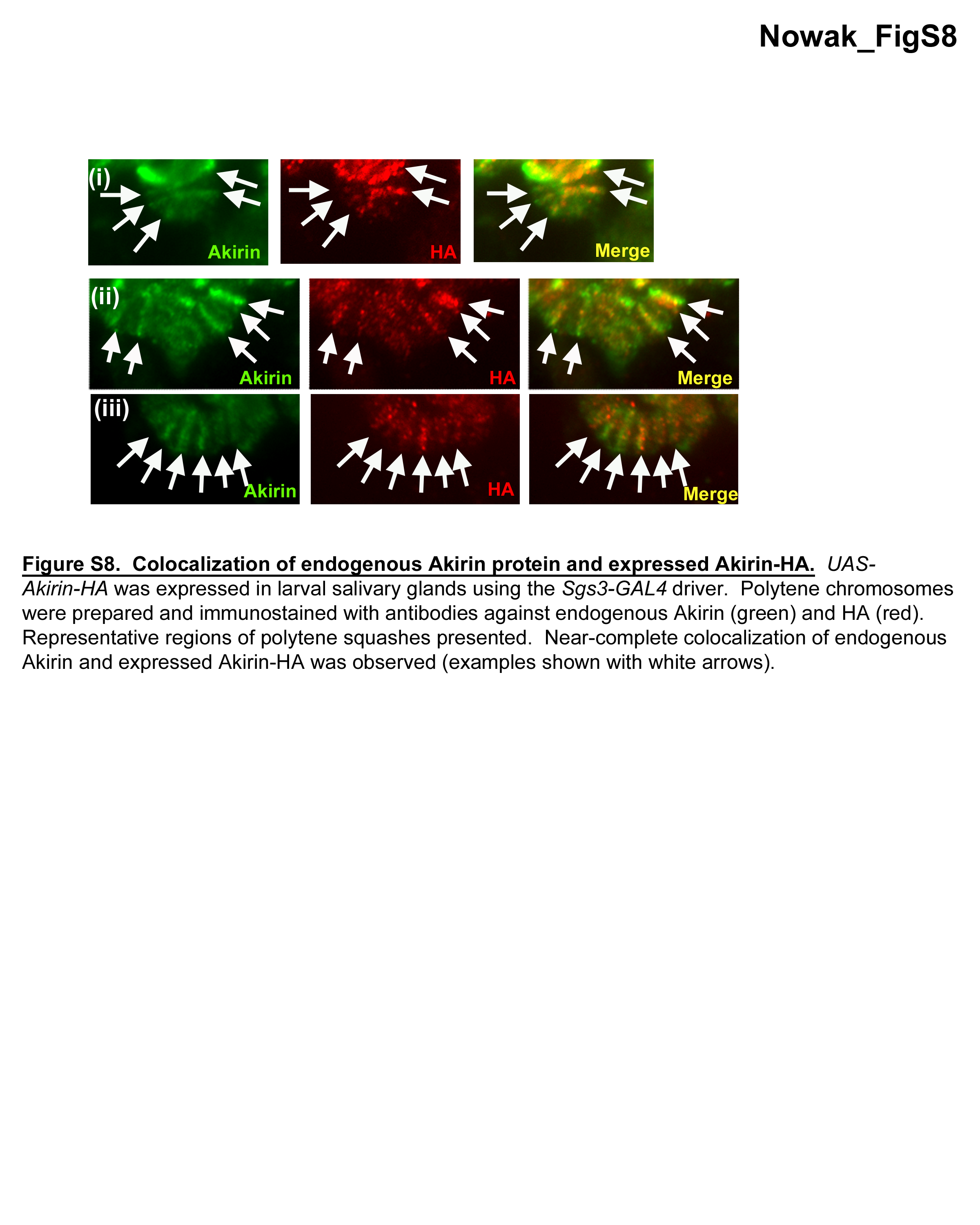

Supplement: Figure S8 — Colocalization of endogenous Akirin protein and expressed Akirin-HA. UAS-Akirin-HA was expressed in larval salivary glands using the Sgs3-GAL4 driver. Polytene chromosomes were prepared and immunostained with antibodies against endogenous Akirin (green) and HA (red). Representative regions of polytene squashes presented. Near-complete colocalization of endogenous Akirin and expressed Akirin-HA was observed (examples shown with white arrows). (TIF) [file pgen.1002547.s008.tif]

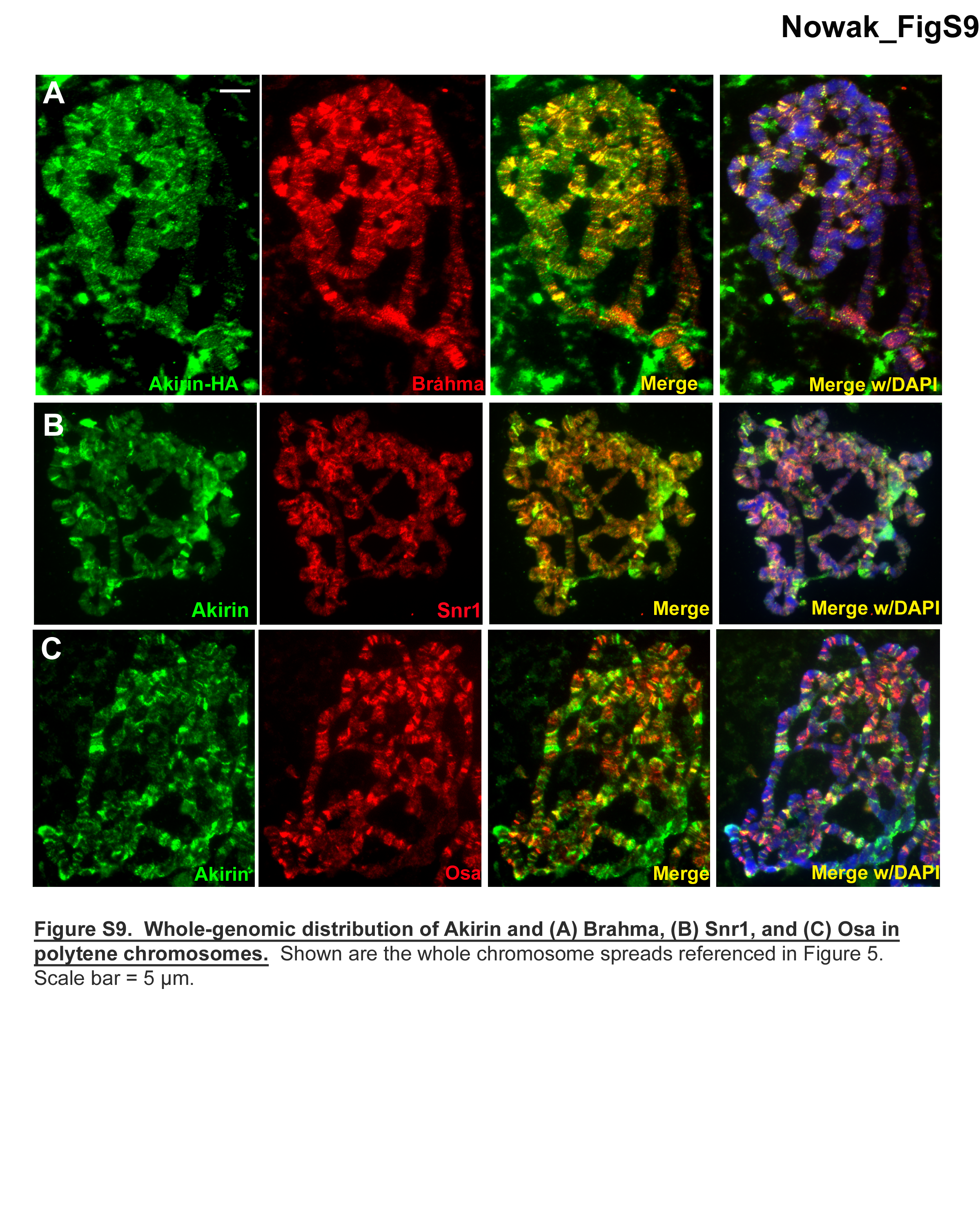

Supplement: Figure S9 — Whole-genomic distribution of Akirin and (A) Brahma, (B) Snr1, and (C) Osa in polytene chromosomes. Shown are the whole chromosome spreads referenced in Figure 5. Scale bar = 5 µm. (TIF) [file pgen.1002547.s009.tif]

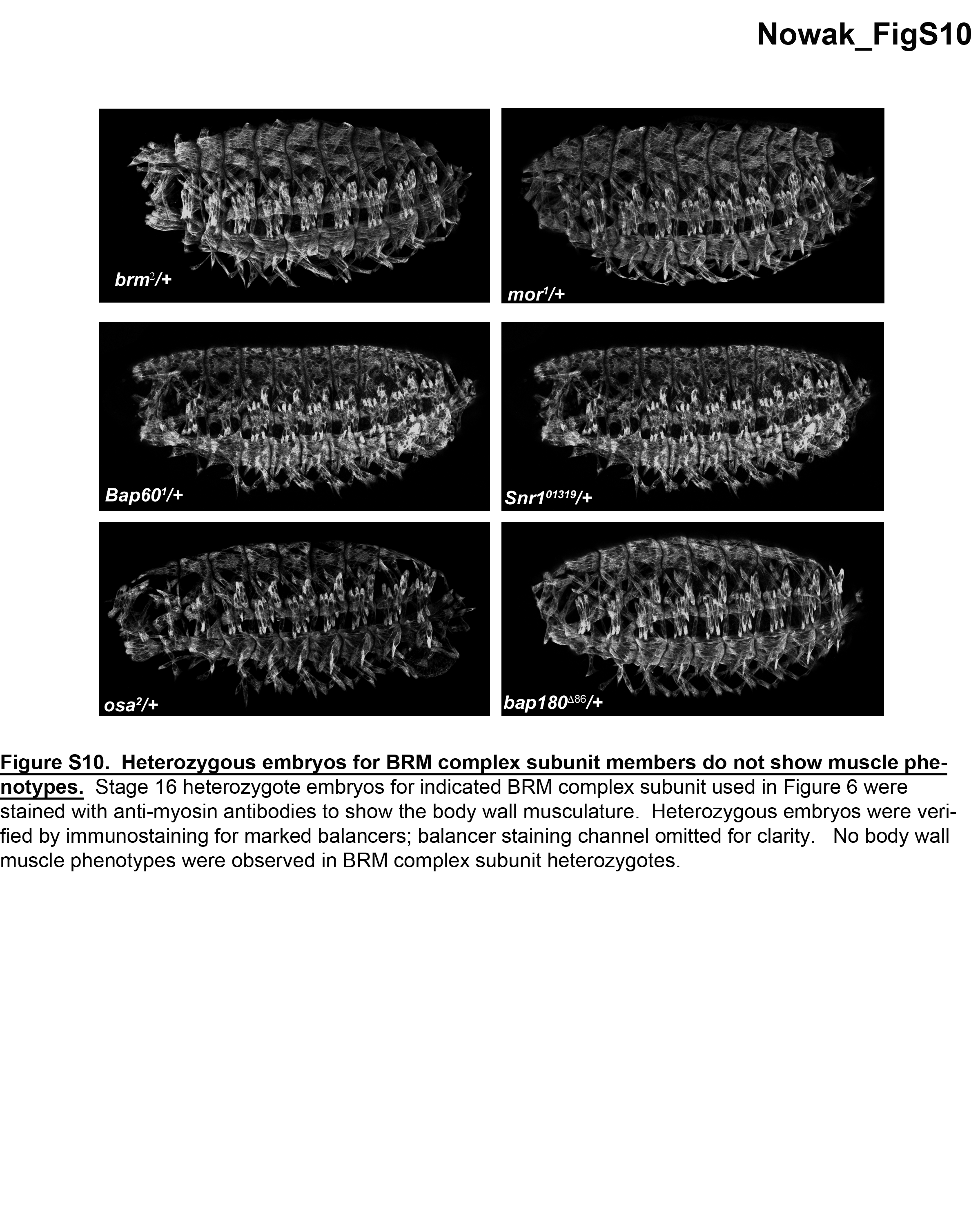

Supplement: Figure S10 — Heterozygous embryos for BRM complex subunit members do not show muscle phenotypes. Stage 16 heterozygote embryos for indicated BRM complex subunit used in Figure 6 were stained with anti-myosin antibodies to show the body wall musculature. Heterozygous embryos were verified by immunostaining for marked balancers; balancer staining channel omitted for clarity. No body wall muscle phenotypes were observed in BRM complex subunit heterozygotes. (TIF) [file pgen.1002547.s010.tif]

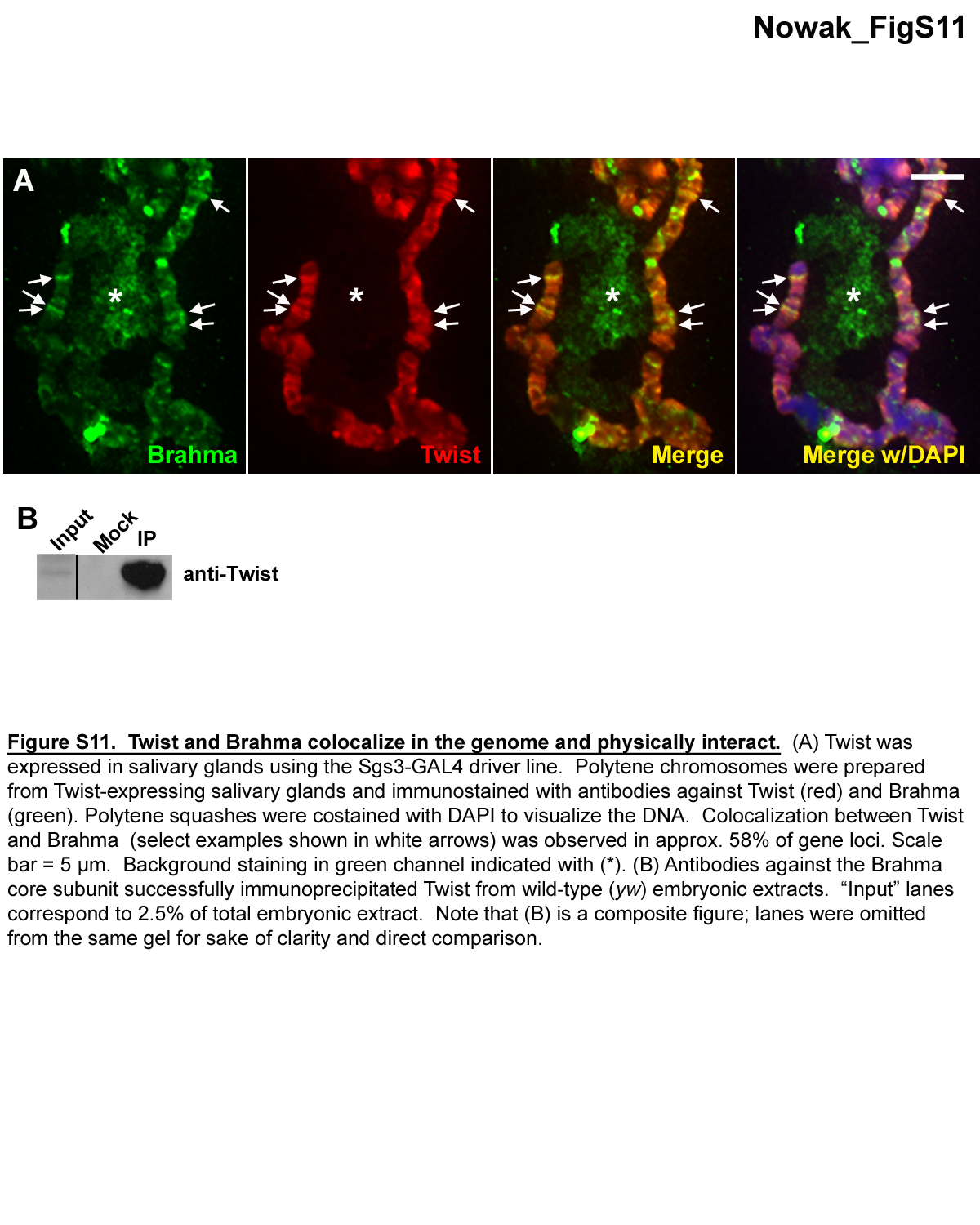

Supplement: Figure S11 — Twist and Brahma colocalize in the genome and physically interact. (A) Twist was expressed in salivary glands using the Sgs3-GAL4 driver line. Polytene chromosomes were prepared from Twist-expressing salivary glands and immunostained with antibodies against Twist (red) and Brahma (green). Polytene squashes were costained with DAPI to visualize the DNA. Colocalization between Twist and Brahma (select examples shown in white arrows) was observed in approx. 58% of gene loci. Scale bar = 5 µm. Background staining in green channel indicated with (*). (B) Antibodies against the Brahma core subunit successfully immunoprecipitated Twist from wild-type (yw) embryonic extracts. “Input” lanes correspond to 2.5% of total embryonic extract. Note that (B) is a composite figure; lanes were omitted from the same gel for sake of clarity and direct comparison. (TIF) [file pgen.1002547.s011.tif]

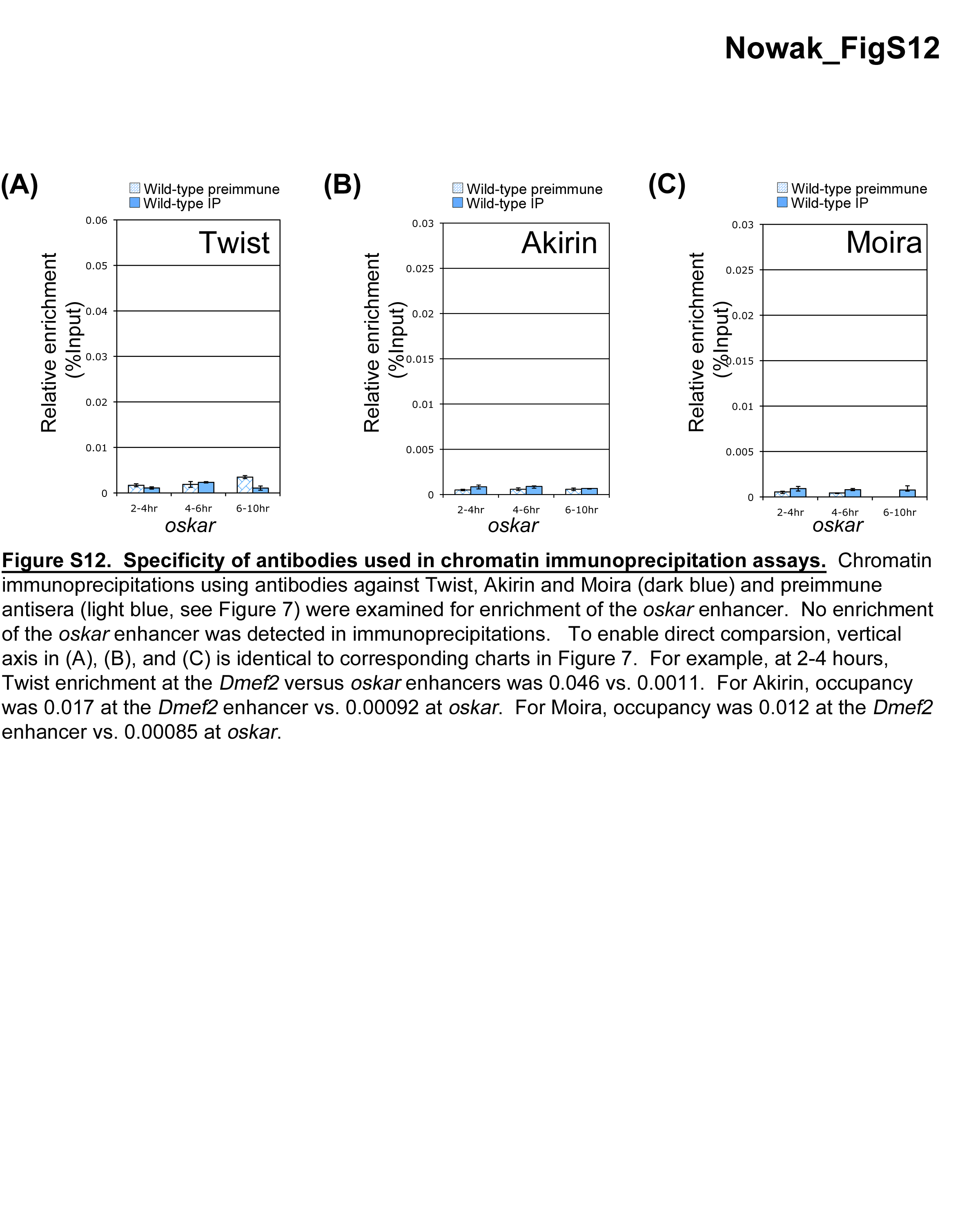

Supplement: Figure S12 — Specificity of antibodies used in chromatin immunoprecipitation assays. Chromatin immunoprecipitations using antibodies against Twist, Akirin and Moira (dark blue) and preimmune antisera (light blue, see Figure 7) were examined for enrichment of the oskar enhancer. No enrichment of the oskar enhancer was detected in immunoprecipitations. To enable direct comparsion, vertical axis in (A), (B), and (C) is identical to corresponding charts in Figure 7. For example, at 2–4 hours, Twist enrichment at the Dmef2 versus oskar enhancers was 0.046 versus 0.0011. For Akirin, occupancy was 0.017 at the Dmef2 enhancer versus 0.00092 at oskar. For Moira, occupancy was 0.012 at the Dmef2 enhancer versus 0.00085 at oskar. (TIF) [file pgen.1002547.s012.tif]
